# Comparative Analysis of Serine/Arginine-Rich Proteins across 27 Eukaryotes: Insights into Sub-Family Classification and Extent of Alternative Splicing

**DOI:** 10.1371/journal.pone.0024542

**Published:** 2011-09-14

**Authors:** Dale N. Richardson, Mark F. Rogers, Adam Labadorf, Asa Ben-Hur, Hui Guo, Andrew H. Paterson, Anireddy S. N. Reddy

**Affiliations:** 1 Department of Bioinformatics and Population Genetics, Universität zu Köln, Köln, Germany; 2 Computer Science Department, Colorado State University, Fort Collins, Colorado, United States of America; 3 Department of Biology, Program in Molecular Plant Biology, Program in Cell and Molecular Biology, Colorado State University, Fort Collins, Colorado, United States of America; 4 Plant Genome Mapping Laboratory, University of Georgia, Athens, Georgia, United States of America; Michigan State University, United States of America

## Abstract

Alternative splicing (AS) of pre-mRNA is a fundamental molecular process that generates diversity in the transcriptome and proteome of eukaryotic organisms. SR proteins, a family of splicing regulators with one or two RNA recognition motifs (RRMs) at the N-terminus and an arg/ser-rich domain at the C-terminus, function in both constitutive and alternative splicing. We identified SR proteins in 27 eukaryotic species, which include plants, animals, fungi and “basal” eukaryotes that lie outside of these lineages. Using RNA recognition motifs (RRMs) as a phylogenetic marker, we classified 272 SR genes into robust sub-families. The SR gene family can be split into five major groupings, which can be further separated into 11 distinct sub-families. Most flowering plants have double or nearly double the number of SR genes found in vertebrates. The majority of plant SR genes are under purifying selection. Moreover, in all paralogous SR genes in Arabidopsis, rice, soybean and maize, one of the two paralogs is preferentially expressed throughout plant development. We also assessed the extent of AS in SR genes based on a splice graph approach (http://combi.cs.colostate.edu/as/gmap_SRgenes). AS of SR genes is a widespread phenomenon throughout multiple lineages, with alternative 3′ or 5′ splicing events being the most prominent type of event. However, plant-enriched sub-families have 57%–88% of their SR genes experiencing some type of AS compared to the 40%–54% seen in other sub-families. The SR gene family is pervasive throughout multiple eukaryotic lineages, conserved in sequence and domain organization, but differs in gene number across lineages with an abundance of SR genes in flowering plants. The higher number of alternatively spliced SR genes in plants emphasizes the importance of AS in generating splice variants in these organisms.

## Introduction

Pre-messenger RNA (pre-mRNA) splicing is a complex and critical molecular process that generates functional mRNA molecules via the precise removal of introns and ligation of exons and is an important gene regulatory step in eukaryotic gene expression [1], [2], [3]. Pre-mRNA splicing is carried out via a macromolecular protein complex known as the spliceosome, which contains five small nuclear ribonucleoprotein particles (snRNPs; U1, U2, U4/U6, and U5) and a large number of auxiliary proteins [around 150 in animals [4], [5]] that act coordinately to catalyze the splicing reaction [6]. Following the discovery that genes are comprised of exons and introns [7], it became evident that a single gene could give rise to multiple alternative mRNA transcript isoforms [8].

Alternative splicing (AS) of pre-mRNA is arguably one of the most important biological processes for expanding the eukaryotic proteome and can help explain the apparent discrepancy between gene content and organismal complexity [9], [10]. AS yields more than one mRNA isoform from a single gene by regulated selection of alternative splice sites [11], which typically give rise to four types of AS events: alternative 5′ splice site choice, alternative 3′ splice site choice, cassette-exon inclusion or skipping, and intron retention [10]. AS not only contributes to an increase in proteomic expansion [9], but also alters protein functionality (gain, loss or reduction in function), localization, and may introduce premature termination codons leading to nonsense mediated decay (NMD) of AS isoforms [11] (and references therein). Recent estimates based on high-throughput studies suggest that 95–100% of all human multi-exon genes undergo AS [12], [13], in contrast to the ∼40% of multi-exon genes estimated to exhibit AS in plants [14], [15], [16], [17].

Given the widespread prevalence of AS in eukaryotic lineages [18], what components contribute to its regulation? One pivotal family of splicing factors has stood out ever since their discovery in the 1990s: the serine/arginine-rich (SR) proteins [19], [20]. The SR proteins were originally classified as a family based on their ability to restore splicing activity to splicing factor deficient cell extracts, their conservation across vertebrates and invertebrates [20], and their recognition by monoclonal antibody mAb104 [21]. Recently, a more precise definition of mammalian SR proteins and unified nomenclature for each protein was proposed [22]. Following that study, plant SR proteins were also redefined and a standardized nomenclature system was adopted for plant SR proteins [23]. All SR proteins have a modular structure consisting of one or two N-terminal RNA recognition motifs (RRMs) and a variable length C-terminal domain rich in serine and arginine residues (the RS domain) [24]. The RRM domains can recognize and bind to a variety of mRNA cis-regulatory elements, albeit with specific yet degenerate RNA binding specificities [24]. The RS domain is required for essential SR protein function, but is intrinsically disordered, meaning that this domain exists in an ensemble of conformations in physiological conditions [25]. However, perhaps because of this disorder, RS domains are able to function as splicing activation domains by contacting the pre-mRNA directly to promote spliceosome assembly [26], [27], [28], foster protein-protein interactions [29], undergo heavy phosphorylation and dephosphorylation (thereby modulating interactions with other proteins or RNA) [30], and contain signals for nuclear localization and nucleocytoplasmic shuttling [31], [32].

Human SF2/ASF (SRSF1) was the first SR protein identified [19], [33], which was followed by the identification of the other classical SR proteins [SC35 (SRSF2), SRp20 (SRSF3), SRp75 (SRSF4), SRp40 (SRSF5), SRp55 (SRSF6) and 9G8 (SRSF7) (reviewed in [34])]. SF2/ASF (and the other SRs listed above) function in constitutive and alternative splicing [34]. SF2/ASF facilitates 5′ splice site recognition by promoting the recruitment of U1snRNP to the 5′ splice site via interactions with U1-70K [29]. SF2/ASF and SC35 both interact with U1-70K and U2AF35 to promote 3′ splice site recognition via recruitment of U2AF65 to the 3′ splice site [35]. Engagement of the tri-snRNP complex U4/U6/U5 in addition to other proteins, including SRs, promotes spliceosome assembly and permits the splicing reaction to occur [36] (and references therein). Besides their roles in constitutive and alternative splicing, SR proteins have also been implicated in mRNA export, RNA stability, nonsense mediated decay (NMD) and translation [36] (and references therein).

SR proteins have been found in all metazoans [20], in lower eukaryotes such as *Schizosaccharomyces pombe* [37] and *Trypanosoma cruzi* [38], and in plants such as Arabidopsis [39], rice [40] and maize [41]. To date, plants possess the most SR proteins of any organism studied, with Arabidopsis encoding 18 SRs and rice encoding 22 [36]. In addition to acting as regulators of AS, SR genes are also alternatively spliced. Recent studies in Arabidopsis indicated a six-fold increase in the SR gene transcriptome (14 SR genes giving rise to 93 distinct AS isoforms) in response to hormones and stresses [42], and extensive coupling of AS isoforms with NMD [43]. Since SR genes are the targets of regulated AS in response to developmental or stress cues, they are most likely targets of multiple signaling pathways and may function as key components in the response to developmental and environmental signals [36].

As SR proteins are prominent players involved in spliceosome assembly, constitutive and alternative splicing of pre-mRNAs including their own transcripts, and are essential for proper gene expression, studying these master regulators in a comparative genomics context would provide insight on SR gene evolution across multiple eukaryotic species. Much of the research focus has been on metazoan SR gene evolution and function, with ample studies conducted in human, drosophila and roundworm (c.f. [34]). However, in the plant kingdom the study of SR proteins and their AS events have either been restricted to a subset of plants e.g., Arabidopsis, rice, moss [44], and maize, pine and *Chlamydomonas* [45], [46], or a subset of SR proteins, e.g., members of the plant specific RS subfamily or the RS2Z subfamily [45]. Therefore, a comprehensive analysis which takes advantage of newly sequenced genomes of photosynthetic and non-photosynthetic eukaryotes to assess the inventory of SR proteins and updated expression data to measure the extent of their AS would contribute to our understanding of the evolution of SR proteins and their importance in generating transcriptome diversity.

By using genome sequence data for phylogenetically diverse eukaryotes, we address a series of questions about plant SR gene content and evolution. Specifically: i) how many sub-families comprise the SR gene family across eukaryotes? ii) do flowering plants have a higher number of SR genes than other eukaryotes? iii) what selective forces are acting upon SR genes? iv) is AS in plant SRs as widespread as in Arabidopsis? v) are SR genes alternatively spliced in all sampled organisms? vi) what are the most prevalent AS event types in SR genes?

To begin addressing these questions, we have mined SR genomic sequences, amino acid sequences and EST/cDNA sequences for 12 photosynthetic eukaryotes and 15 non-photosynthetic eukaryotes from publicly available databases. Tentative SR gene inventories for 10 of the 12 photosynthetic eukaryotes and 12 of the 15 non-photosynthetic eukaryotes were determined in this study. We show that the SR gene complement from these organisms falls into five major groups, which can be further separated in to 11 sub-families. Furthermore, it appears that it is a general characteristic of photosynthetic organisms to possess on average a larger inventory of SR genes than non-photosynthetic organisms. We go on to show that most SR genes in photosynthetic eukaryotes are under purifying selection, that paralogous SR genes in some photosynthetic organisms are divergently expressed throughout development and that alternative splicing of SR genes is a common phenomenon shared by the majority of eukaryotes analyzed here.

## Results

### SR genes form between five and eleven sub-families

**Figure 1:**
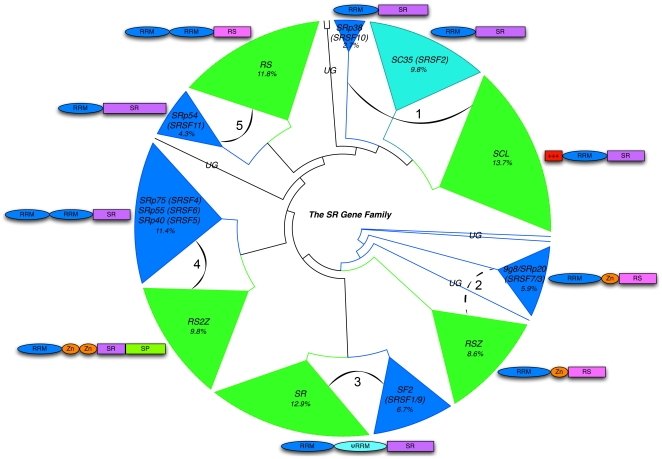
Condensed SR gene family tree. Schematic representation (from FigTree [87]) of the sub-family relationships among 272 SR genes from the organisms sampled in this study. The numbered curved lines indicate the major groupings. Domain organization is presented adjacent to each clade. Green clades represent plant-enriched or plant-specific sub-families, whereas blue clades represent non-photosynthetic organisms. The turquoise SC35 clade denotes the mixture of plant SC members and non-plant SRSF2 members. The sum of these clades will yield 11 distinct sub-families. Taxa grouped into plant-enriched families that are non-photosynthetic are indicated in red. Species prefixes are as follows: *Gm, Glycine max; Pt, Populus trichocarpa; At, Arabidopsis thaliana; Vv, Vitis vinifera; Zm, Zea mays; Sb, Sorgum bicolour; Os, Oryza sativa; Sm, Selaginella moellendorfii; Pp, Physchomitrella patens; Cr, Chlamydomonas reinhardtii; Cv, Chlorella vulgaris; Cm, Cyanidioschyzon merolae; Hs, Homo sapiens; Mm, Mus musculus; Gg, Gallus gallus; Xt, Xenopus tropicalis; Dr, Danio rerio; Br, Branchiostoma floridae; Ci, Ciona intestinalis; Dm, Drosophila melanogaster; Ag, Anopheles gambiae; Aa, Aedes aegyptii; Ce, Caenorhabditis elegans; Nc, Sp, Schizosaccharomyces pombe; Dd, Dictyostelium discoideum; Pf, Plasmodium falciparum; Ps, Phytopthora sojae*. UG, ungrouped.

We acquired SR genomic, EST/cDNA and amino acid sequences for 27 different eukaryotic species that span a diverse array of lineages (Figure S1 and Table 1). We retrieved the sequences for 272 SR genes, and used the amino acids of the RRM domains to construct a multiple alignment for gene-tree reconstruction (see methods). We consolidated the scattered inventory of SR genes from multiple organisms into robust sub-family classifications. Using two maximum likelihood methods and one parsimony method, we inferred that there are at least five major SR gene sub-families: SCL and SC35, RSZ and 9g8 (SRp20/SRSF7/3), SR and SF2 (SRSF1/9), SRp40/55/75 (SRSF4/6/5) and RS2Z, and RS and SRp54 (SRSF11) (Figure 1). However, based on unique domain structures and gene-tree support values (Figure 1 and Figures S3, S4, S5, S6, S7), the five major sub-families can be further partitioned into 11 distinct sub-families. Maximum likelihood scores and domain organization were used in dividing SRs into 11 subfamilies.

**Table 1:** The 27 organisms, their SR repertoire and databases used.

Organism	#SRs	Reference	Database
*Glycine max*	25*	EH	[89]
*Populus trichocarpa*	20	EH	[89]
*Arabidopsis thaliana*	18	[70]	[90]
*Vitis vinifera*	9	EH	[89]
*Zea mays*	22	EH	[91]
*Sorghum bicolor*	19*	EH	[89]
*Oryza sativa*	22	[44]	[92]
*Selaginella moellendorffi*	3*	EH	[89]
*Physcomitrella patens*	10	EH	[89]
*Chlamydomonas reinhardtii*	5	EH	[89]
*Chlorella vulgaris*	3*	EH	[89]
*Cyanidioschyzon merolae*	2	EH	[93]
*Homo sapiens*	11	[64]	[94]
*Mus musculus*	10	EH	[94]
*Gallus gallus*	10	EH	[94]
*Xenopus tropicalis*	11	EH	[94]
*Danio rerio*	14	EH	[94]
*Branchiostoma floridae*	11	EH	[95]
*Ciona intestinalis*	8	EH	[95]
*Drosophila melanogaster*	7	[65]	[96]
*Anopeheles gambiae*	6	EH	[94]
*Aedes aegypti*	6	EH	[94]
*Caenorhabditis elegans*	7	[66]	[97]
*Schizosaccharomyces pombe*	2	[37]	[98]
*Dictyostelium discoidum*	2	EH	[98]
*Plasmodium falciparum*	3	EH	[99]
*Phytophthora sojae*	3	EH	[95]

Organisms are listed according to their groupings in Figures S2, S3, S4, S5, S6, S7.

*, These organisms may have more SRs than listed due to the exclusion of sequences that did not begin with methionine residues; EH, Extracted here.

SR genes from 12 photosynthetic eukaryotes contributed to roughly 62% of the five major groupings observed (green clades in Figure 1 and Figures S2, S3, S4, S5, S6, S7). About 2% of the SR genes were unresolved in the gene tree analyses, which included taxa from the single celled eukaryotes *C. reinhardtii*, *C. elegans*, *S. pombe*, *B. floridae* and *P. sojae*. Sub-families were labeled according to pre-existing family nomenclature (SC35 (SRSF2), SCL, RS, SR, RS2Z, 9G8/SRp20 (SRSF7/SFSR3), SF2 (SRSF1), or by prominent SR genes populating a clade (SRp38 (SRSF10), SRp40 (SRSF5), SRp55/75 (SRSF6/SRSF4), RSZ and SRp54 (SRSF11). Using the nomenclature guidelines outlined in [23], we re-named all photosynthetic SR proteins accordingly and consistently (see parenthetical labels in Figures S2, S3, S4, S5, S6, S7). It should be noted that the clades RSZ and SR (consisting of only photosynthetic eukaryotes) are considered to be orthologous to the 9G8 and SF2/ASF sub-families, respectively (groups 4 and 5 in Figure 1) [36].

### SC35 (SRSF2) is likely an ancient SR gene

SC35 is present within eight of the photosynthetic organisms and all of the bilateral metazoans, and *C. merolae*, the ancient red alga believed to have originated prior to the last common ancestor among plants, animals and fungi [47]. However, it is absent from fungi and lower eukaryotes (Figure S3). The lack of SC35 in the fungi, lower eukaryotes and some of the multi-cellular plants is surprising, because SC35 is one of the core SR proteins that participates in 5′ and 3′ splice site recognition and interacts with U170-K and U2AF35 [47]. However, in the photosynthetic eukaryotes it is likely that other SR proteins perform similar functions to SC35 thereby mitigating its loss in these genomes.

### Some clades are vastly expanded in plants, with three of them plant-specific

**Figure 2:**
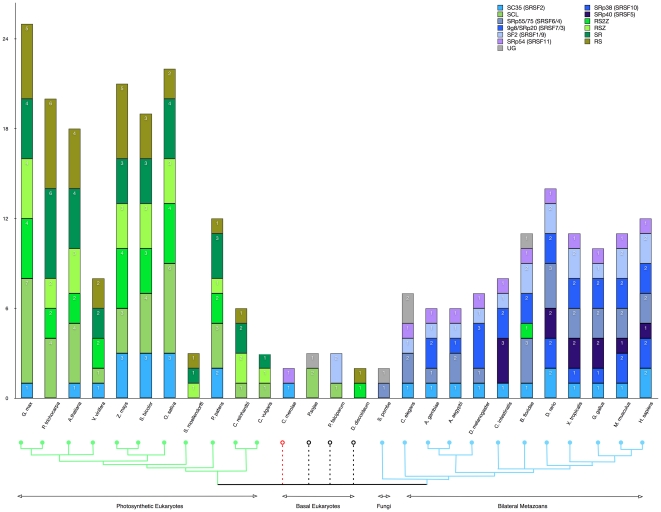
Sub-family classification of SR genes. Based on the trees presented in Figure 1 and Figure S2, we plotted the SR clades by organism. The inferred taxonomic grouping from Figure S1 is plotted below the bar chart and the number of SR genes per family is indicated by color codes as well as value labels. Note: SRp40 (SRSF5) is shown in its own grouping to highlight the divergence of the insects from the predominantly mammalian SRp55/75 (SRSF6/4) clades.

Some clades contributed to the generally larger number of SR genes found in photosynthetic eukaryotes: RS, SR, RSZ, RS2Z and SCL (with RS, RS2Z and SCL being plant-specific; Figure 2). The RS sub-family (31 members) is unique to photosynthetic eukaryotes, except for a single SR protein from *D. discoideum* that also grouped into this family (Figure S4). Though this *D. discoideum* sequence possesses two RRMs, which is characteristic of RS family members, its relatively long branch (0.93, and indicated in red in Figure S4), long full-length sequence (737 aa) and modest bootstrap support values (36% RAxML, 23% Garli) call its grouping within the plant-specific RS clade into question. Nevertheless, the hypothesis that this protein is indeed a distant member of the RS sub-family cannot be unequivocally disregarded. Bearing this in mind as a singular exception, the members of the RS sub-family are only present in the embryophyta and absent in the algal species, except for *C. reinhardtii*. Among the dicotyledenous plants, *P. trichocarpa* possesses the most RS sub-family members (six), whereas *V. vinifera* possesses the fewest (two) (Figure 2). Interestingly, the low number of RS members in rice was not a characteristic feature among monocots (c.f. *Z. mays*, Figure 2).

Another expanded plant-specific grouping is the single RRM, two-zinc knuckle family, RS2Z (25 members) (Figure 2 and Figure S5). RS2Z formed a sister group with SRp40/55/75, but its unique domain structure is found only in photosynthetic SR genes (Figure 1). In contrast to the RS sub-family, RS2Z family members are restricted to the monocot and dicot lineages. In dicots, *G. max* has the most members (four) compared to Arabidopsis, *P. trichocarpa* and *V. vinifera*, which only have two members each. Each of the monocotyledonous organisms has four members (one member from *S. bicolor* was not officially counted because it did not pass our selection criteria; see methods). Notably, one of the RS2Z members from *G. max*, GmRS2Z21 (underlined in Figure S5), does not possess the dual zinc finger motifs characteristic of this sub-family and may be excluded from this sub-family. This could be an error in genome annotation; however, GmRS2Z21 is relatively well supported by all three tree-searching methods (64% RAxML, 62% Garli, 60% parsimony).

Interestingly, two non-photosynthetic SR genes (one from *D. discoideum* and one from *B. floridae*) grouped into the RS2Z sub-family with moderately weak support values and relatively long branches (DdB0233308 0.93 and Br125053 0.67; bootstrap support: 13% RAxML, 10% Garli, 27% parsimony) (Figure S5). The *D. discoideum* sequence possesses two zinc fingers and the *B. floridae* sequence possesses one zinc finger. Because of the questionable support values, sequences from other organisms related to these are needed to determine if RS2Z is an ancient SR gene sub-family that was later lost in the Euteleostomi.

The largest plant-specific sub-family is the SCL family (containing a single RRM domain) with 37 members (Figure 2, Figure S3). The family is present within the dicots, monocots, *P. patens* and the green algae, but absent from the remaining photosynthetic eukaryotes. *G. max* possesses the most SCL proteins (seven) among dicots, whereas rice possesses the most among the monocots (six). Interestingly, the bilateral metazoan conserved SRp38 (SRSF10) sub-family was a close sister group to the SCL sub-family (bottom clade in Figure S3). This similarity was previously acknowledged [36] as SRp38 members are splicing repressors. However, whether or not SCL proteins function as splicing repressors is an unanswered question. Strikingly, three sequences from *P. sojae*, a plant pathogenic stramenopile, also grouped into the SCL sub-family, albeit with either long branches, poor bootstrap support or both (red taxa in Figure S3). Not only does this grouping of stramenopile sequences hint at the possibility of the SCL sub-family not being truly plant-specific, but also raises speculation into whether or not this evolutionary similarity is coupled to the coevolution of pathogenicity.

The remaining two sub-families, SR (33 members, Figure S6) and RSZ (23 members, Figure S5) are not plant-specific per se, but are orthologous to SF2/ASF (SRSF1) (Figure S6) and 9G8/SRp20 (SRSF3) (Figure S7), respectively. SR and RSZ members are greatly enriched in plants. *P. trichocarpa* contains six members of the SR sub-family, the most of any photosynthetic organism (Figure 2). As mentioned previously, SR is present in all photosynthetic lineages except for *C. merolae*, suggesting that this family was probably derived after the divergence of the red algae from plants and animals, but prior to the split of plants from animals. A similar situation is observed with respect to the RSZ sub-family: it is present in all photosynthetic eukaryotes (orthologous 9G8/SRp20 is present in all bilateral metazoans, as well), but absent in *C. merolae*, fungi and the other basal eukaryotes (dashed black lines in Figure 2).

### Five SR clades are conserved across bilateral metazoans

Clades SRp54 (SRSF11), SF2 (SRSF1), 9G8/SRp20 (SRSF7), SRp40/55/75 (SRSF5/SRSF6/SRSF4) and SC35 (SRSF2) are broadly conserved across the bilateral metazoans, with the exception that SRp55/75 (top blue clade in Figure S5) and SRp38 (bottom blue clade in Figure S3) are only observed in the Euteleostomi (*D. rerio*, *X. tropicalis*, *G. gallus*, *M. musculus* and *H. sapiens*) [Figure 2]. This suggests that SRp40 diverged from SRp55/75 after the split between insects and mammals and that SRp38 was probably lost in the insect lineages. Interestingly, *C. merolae* has a single member of the SRp54 sub-family that has moderate ML bootstrap support (29% RAxML) and branch length (0.73) [Figure S4]. Therefore, a likely scenario is that SRp54 evolved prior to the divergence of plants from animals, but underwent several losses in multiple lineages. The 9G8/SRp20 sub-family also appears to have an early derivation given the sister grouping of an SR protein from *P. sojae* (Figure S7) as well as the zinc finger domain being shared between the plant-enriched RSZ sub-family.

### Basal eukaryotes have the fewest SR sub-families

The lowest number of SRs was found in the basal eukaryotes (*P. sojae*, *P. falciparum*, *D. discoideum*), the algal species (*C. reinhardtii, C. vulgaris and C. merolae*) and the fission yeast, *S. pombe* (Figure 2 and Table 1). Each of these organisms, except for *C. merolae* and *D. discoideum* contained at least one SR protein that was not resolved in our gene tree analyses (Figures 1, 2 and Figure S2). The low number of SR genes in these organisms is likely a reflection of organism complexity, the degree of multi-intron containing genes within a genome (e.g., only 43% of genes in *S. pombe* contain introns, of those only 25% have more than one intron, [48]) and limited alternative splicing of pre-mRNAs.

### RNA binding motifs are variable within RRM regions

**Figure 3:**
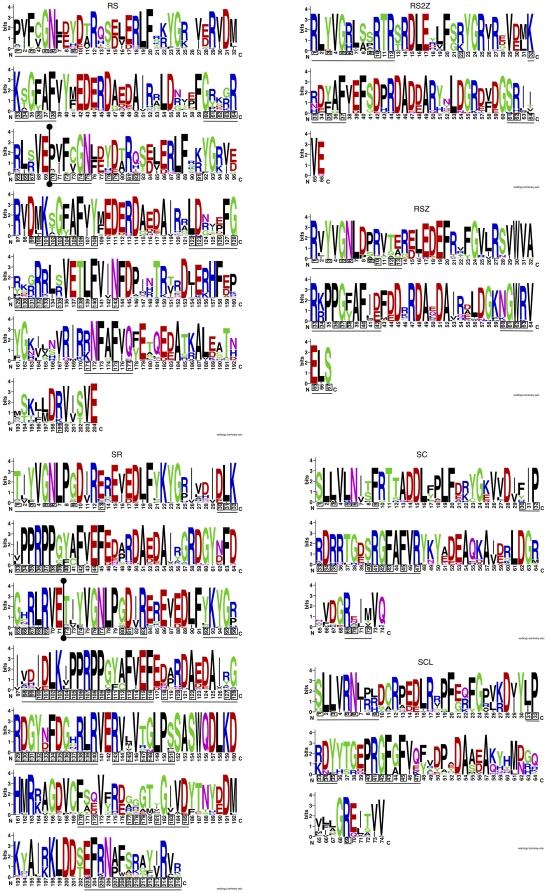
RRM domain web logos for plant-enriched sub-families. Web logos were created for each of the plant-enriched sub-families and putative RNA binding residues are indicated by boxes and underlined. Web logos were created by using the web logo server [88] and binding residues were predicted using the PiRanhA webserver [49], [50]. In the cases of the RS and SF2(p) sub-families, the demarcation of RRMs is indicated by a vertical bar with circular endpoints.

In order to ascertain which residues within the highly conserved RRM regions of SR genes are involved in binding to mRNA molecules, we used the PiRaNhA machine learning web server to predict potential RNA binding residues [49], [50]. PiRaNha uses various amino acid sequence features, such as residue interface propensity, predicted residue accessibility and residue hydrophobicity to predict RNA-binding residues. Ten randomly selected RRM sequences from each plant-enriched grouping (RS, RSZ, RS2Z, SR, SC and SCL) were analyzed using the PiRaNhA webserver (Figure 3). We used ten sequences because each of the plant-enriched clades had at least ten members. Boxes indicate potential amino acid residues implicated in RNA binding and motif regions are underlined in Figure 3. Interestingly, the majority of binding regions include highly variable positions within the RRM. Often, putative RNA binding residues are variable yet surrounded by a few highly conserved amino acid positions (Figure 3). In all analyzed clades, the first nine to 13 amino acids of the RRM are implicated in RNA binding and in the case of the RS and SR sub-families, the second RRM region contains many more RNA binding regions. Previously, the structure of hnRNP A1, an antagonist to the SC35 (SRSF2) and SF2/ASF (SRSF1) SR splicing factors was determined [51]. The RNP-1 sub-motif of hnRNP A1 (*RGFgFvty*) was shown to bind single stranded DNA and is highly similar to the *RDFAFVR* motif of the SC sub-family (middle-right panel, residues 41–47 in Figure 3). These similar binding motifs could explain the antagonistic nature of these proteins. Furthermore, predictions of RNA binding residues by PiRaNhA provide a suitable starting point for site-directed mutagenesis experiments in RRMs of plant-enriched SR proteins.

### SR genes in photosynthetic eukaryotes are mostly under purifying selection

As genome duplication has played a pivotal role in plant evolution, we investigated the impact of whole genome duplication on SR genes in the flowering plant lineages we sampled. Using the plant genome duplication database (http://chibba.agtec.uga.edu/duplication/) and following previously described methods [52], orthologous SR genes (identified by considerations of neighboring gene content) from Arabidopsis, *G. max*, rice, poplar, *S. bicolor* and *V. vinefera* were evaluated for their substitution rates, specifically, the ratio of the rate of non-synonymous to synonymous substitutions (*K*_a_/*K*_s_). Of the 132 orthologs analyzed from these species, only six genes (SbSR32a, OsSR33a; SbSR32a, ZmSR30a; SbSC32, OsSC34; SbSC32, ZmSC30a) showed (*K*_a_/*K*_s_) ratios greater than 0.9 (red crosses in Figure S8), which is indicative of positive selection acting upon these genes. However, the great majority of SR genes (126) appear to be evolving under purifying selection and suggests that new substitutions in SR genes are most likely deleterious and would compromise their biological efficacy in protein-RNA/protein-protein interactions.

### SR paralogs in photosynthetic eukaryotes are expressed at different magnitudes

To further investigate the influence of gene duplication in the SR gene family, we analyzed expression data for paralogous pairs in Arabidopsis, rice, maize and *S. bicolor*. For Arabidopsis, paralogous SR genes were determined by their groupings in Figures S3, S4, S5, S6, S7, and by referring to [53], whereas paralogy for the remaining plant species was based solely on their groupings in Figures S3, S4, S5, S6, S7. Expression data for various developmental stages was extracted using Genevestigator [54] and plots were generated for the paralogs.

**Figure 4:**
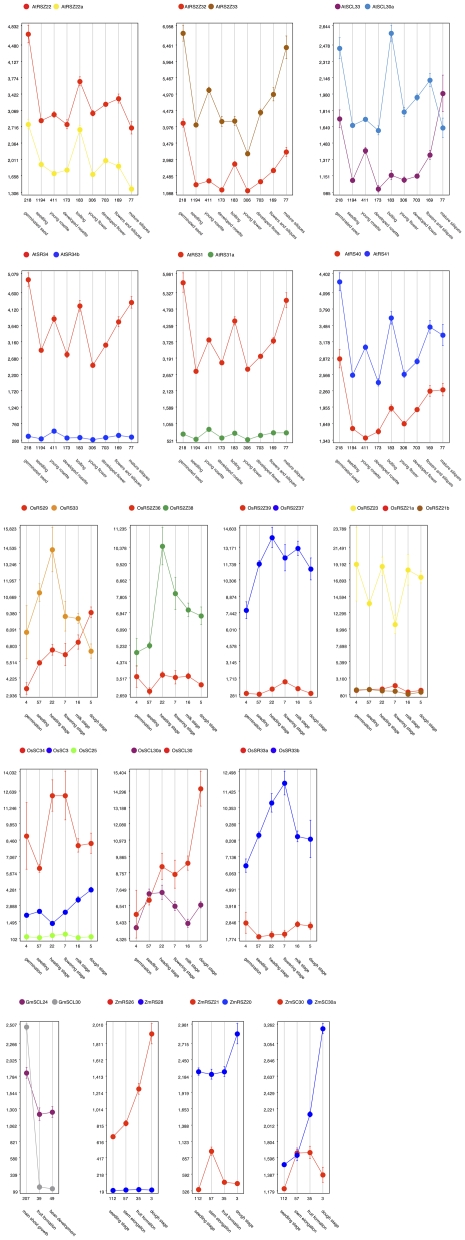
Differential expression of SR gene pairs. Gene expression data for various developmental stages were taken from the Genevestigator database [54] and plotted for each of the six pairs of paralogous SR genes. In some cases (in rice), there were three paralogs included. The numbers below the x-axis indicate the number of microarray experiments that underlie the average intensity value plotted on the y-axis.

In Arabidopsis, there are six SR gene pairs and in every case in each developmental stage, none of the paralogs were expressed at the same levels (Figure 4, top six panels). On average, the level of gene expression was around 1.5–2 times greater for one of the two genes in a pair, and sometimes as large as 7–12 times (see AtSR34-AtSR34b and AtRS31-AtRS31a in Figure 4). By contrast, the remaining Arabidopsis SR genes that do not exist as gene pairs have overlapping expression patterns (Figure S9). Note that AtSCL28 and AtSCL30 can be considered as a gene pair according to Figure S3, but since they were not found in [53], we chose to conform to the results presented in [53].

The pattern observed for the six Arabidopsis paralogs was also evident in rice, maize and soybean (lower panels in Figure 4). There was one case of similar expression magnitudes, during the stem elongation stage in maize for ZmSC30a and ZmSC30.

### Alternative splicing of SR genes is widespread

**Figure 5:**
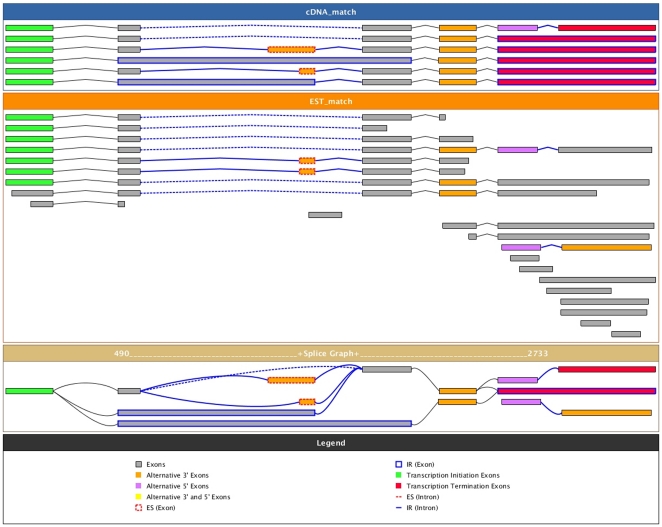
Example splice graph for AtSCL33. Shown here is a typical splice graph from which AS event counts are taken. Full-length cDNAs are shown in the top-most panel, EST matches in the middle and the resultant splice graph is in the lower panel.

**Figure 6:**
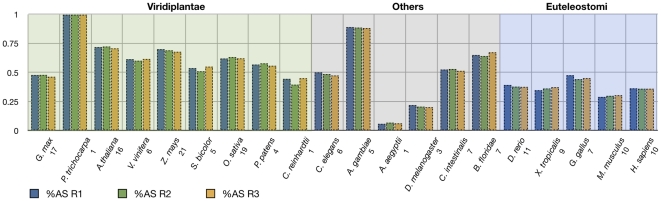
EST/cDNA normalized %AS. As detailed in the methods, we ran 100 trials in triplicate in order to compare alternative splicing evidence between SR genes from different organisms. The organisms are arranged from the Viridiplantae (green shaded area), to “other eukaryotes” (grey shaded area) and finally to the Euteleostomi (blue shaded area). Numbers below the taxon names indicate the number of SR genes that had at least 15 ESTs/cDNAs necessary for the normalization procedure. R1, R2 and R3 correspond to individual runs (100 trials each) of the triplicate series.

The next major component to our analysis of SR genes in eukaryotes was to assess the extent of alternative splicing (AS) among the organisms with sufficient EST/cDNA data. Of the 27 eukaryotes that were included in our phylogenetic analysis, 20 had enough ESTs to be analyzed in our AS pipeline (Table 2; and see methods and online material: http://combi.cs.colostate.edu/as/gmap_SRgenes for a description of the pipeline and resultant splice graphs). An example splice graph for AtSCL33 from which our AS event counts were based is presented in Figure 5. While there were 20 organisms with sufficient expression information, the raw number of ESTs/cDNAs was highly variable between species (Figure S12). Therefore, we imposed a normalization procedure for measuring the extent of AS so that organisms would be comparable, similar to that of [18]. We executed 100 resampling trials in triplicate of our AS pipeline requiring any given gene to have at least 15 ESTs/cDNAs. This procedure limited our dataset substantially, but conferred the ability to make comparisons across species. The non-normalized AS graphs are accessible from the website listed above and the non-normalized fraction of genes undergoing AS is presented in Table 2. Normalized fractions of AS for the three independent replicates are depicted in Figure 6. We observed negligible variance across each of the runs for most of the species, but it should be noted that some species have low sample sizes of between 1–5 SR genes (due to the requirement that a gene have at least 15 ESTs/cDNAs for consideration). Bearing this in mind, the 100% AS of the single *P. trichocarpa* SR gene should not be considered reflective of the extent of AS in this organism's SR genes. Excluding those organisms that had only a single SR gene with at least 15 ESTs/cDNAs, all photosynthetic organisms (green shaded box in Figure 6) had greater than 50% of their SR genes undergoing AS, in contrast to the Euteleostomi (blue shaded box in Figure 6) that had AS percentages ranging from 30%–48%, while the “other” organisms had a much more variable range of % AS (grey shaded box in Figure 6).

**Table 2:** Alternatively spliced SR genes.

Organism	Genes with AS	Total genes	Fraction
*Glycine max*	17	25	0.65
*Populus trichocarpa*	8	20	0.40
*Arabidopsis thaliana*	16	18	0.84
*Vitis vinifera*	6	9	0.66
*Zea mays*	21	22	0.95
*Sorghum bicolor*	11	19	0.55
*Oryza sativa*	20	22	0.83
*Physcomitrella patens*	8	10	0.62
*Chlamydomonas reinhardtii*	2	5	0.29
*Danio rerio*	12	14	0.86
*Xenopus tropicalis*	9	11	0.82
*Gallus gallus*	7	10	0.70
*Mus musculus*	10	11	0.91
*Homo sapiens*	11	12	0.92
*Ciona intestinalis*	7	8	0.88
*Branchiostoma floridae*	9	11	0.82
*Caenorhabditis elegans*	6	7	0.86
*Anopeheles gambiae*	5	6	0.83
*Drosophila melanogaster*	3	7	0.43
*Aedes aegypti*	1	6	0.17

This table contains the non-normalized AS counts from our AS pipeline. Organisms are listed according to their phylogenetic grouping. Though members of the SR45 sub-family were not included in our final gene-tree analyses, we nevertheless analyzed these genes for AS.

We also measured the normalized average type of AS event, among five AS event types (IR, intron retention; SE, skipped exon; Alt 3′, alternative 3′ AS; Alt 5′, alternative 5′ AS; and Alt B, both 3′ and 5′ AS) per gene (Figure S10). Again, gene sample sizes should be taken into consideration when any comparisons are made and special attention given to those organisms that have extremely low sample sizes (i.e., *P. trichocarpa*, *C. reinhardtii*, *A. aegyptii*). Beginning with the Viridiplantae, Arabidopsis and maize had the highest incidence of intron retention events, with an average ranging from 0.84–1.94 events per SR gene (green shaded box in Figure S10). *V. vinifera*, Rice and *G. max* had the next highest incidence of IR, with *P. patens* having zero IR events but the highest average number of skipped exons (2.74 per SR gene) among all sampled organisms. Based on the available data, IR is not the most prevalent AS type among all the Viridiplantae. Instead, Alt 3′, Alt 5′ and SE events appeared to be just as prevalent, and in some cases more prevalent (*G. max*, *V. vinifera*, *S. bicolor*, *O. sativa*, *P. patens*) than IR events.

Regarding the Eutelostomi (blue shaded box in Figure S10), Alt 3′ AS events were generally the most prevalent followed by SE events and then Alt 5′ or IR AS events. *D. rerio* was the exception, with IR being the most prevalent form of AS. This pattern is similar to what was observed in the Viridiplantae in the sense that there was no clearly preferable and broadly shared AS event type. Considering the final group of organisms (grey shaded box in Figure S10), we observe considerable variance in average AS event types, as in the Viridiplantae and Euteleostomi. Some organisms had a higher number of IR events (*C. intestinalis*), whereas others had a higher number of SE events (i.e., *C. elegans* [0.64] and especially *B. floridae* [1.69]).

Finally, we observed that in most organisms, Alt 3′ AS (orange bars in Figure S10) was more prevalent than Alt 5′ AS (purple bars in Figure S10) and that the simultaneous AS of the 3′ and 5′ ends of introns was the least prominent AS event type (yellow bars in Figure S10).

### AS event types vary by sub-family

We next investigated how the percentage of AS and AS event type differed across the various SR sub-families. Using the classifications obtained from our gene tree analyses, the normalized measurements of family-wise %AS were calculated (Figure S11A). All photosynthetic sub-families (green shaded box in Figure S11A) were observed to have between 57%–88% of their SR genes experiencing some type of AS, in contrast to the non-photosynthetic sub-families (blue shaded box in Figure S11A) where the range was between 40%–54%.

For each sub-family, we also calculated the normalized AS event type counts (Figure S11B). As was mentioned above, the occurrence of both Alt 3′ and Alt 5′ (Alt B) splicing of an intron was the least prevalent type of AS event and was also evident in the family-wise comparisons (yellow bars in Figure S11A). The highest average number of Alt B events was observed in the RSZ sub-family (0.17 events per SR gene), followed by 9G8/SRp20 (SRSF7) and SRp54 (SRSF11) (0.09 events per SR gene, respectively). The sub-family with the highest amount of IR events was the plant-specific RS group (0.83 events per SR gene), whereas the family with the lowest amount of IR was SRp38 (SRSF10) (0.08 events per SR gene). Note that as the graph transitions into non-plant enriched sub-families (blue shaded area in Figure S11B), there was a tendency for the incidence of IR to decrease while SE events increased. The plant-specific SCL, RS and plant-enriched SR sub-families had SE events ranging from 0.46 to 0.69 events per SR gene, whereas the other plant-enriched sub-families had much less SE events. Additionally, as previously stated, in nearly all sub-families, the incidence of Alt 3′ AS was more frequent than Alt 5′ AS.

## Discussion

### The SR gene family is large and diverse

There are five major SR groups (Figure 1), which can be further divided into at least 11 sub-families. Five of these sub-families are extensively populated by photosynthetic eukaryotes (RS, RSZ, RS2Z, SCL and SR), six sub-families are highly populated by metazoans (9G8/SRp20 (SRSF7), SRp38 (SRSF10), SRp40 (SRSF5), SRp55/75 (SRSF6/SRSF4), SF2 (SRSF1) and SRp54 (SRSF11), and a single sub-family shares members from both metazoans and plants (SC35/SFSR2) along with a few ungrouped sequences (Figure 1). Interestingly, the ungrouped sequences are primarily from the unicellular eukaryotes and their failure to fall into specific sub-families/clades may be a reflection of their unique life histories or extensive sequence divergence. For example, putative SR proteins from the fission yeast, *S. pombe* and *P. sojae* fall into questionable sister groupings either adjacent to SRp38 (SRSF10) or sister to the 9G8/SRp20 (SRSF7) sub-family, respectively, with either long branches (in the case of SpSRp1) or lack of additional characteristic sub-family domains, such as the zinc finger domain (in the case of Ps136493). However, in a previous study, the two yeast proteins, SRp1 (Ungrouped) and SRp2 [SRp55/75 (SRSF6/SRSF4)] were shown to interact with each other and that their interactions were regulated by phosphorylation, hinting at a possible role in regulation of splicing in the 25% of multi-intronic genes of this organism [37]. Unfortunately, in the previously mentioned study, there were no experiments conducted on alternative splicing. Furthermore, to date, there have not been any reports of alternative splicing in *S. pombe* [55]. Therefore, it is plausible to consider that SR genes in basal unicellular eukaryotes perform rudimentary functions in regulated constitutive splicing. However, if we consider a recent report on the oomycete plant parasite, *P. sojae*, of which two of its three SR genes were resolved into the plant-specific SCL sub-family in the gene-tree analyses (Figure S3), there have been reported incidences of alternative intron processing in family 5 endoglucanase transcripts [56]. It seems that alternative splicing in these organisms is a rare occurrence (neither of these organisms had EST/cDNA data to support AS in their SR genes), and instead, these SR genes might represent ancient prototypical SR genes that were either lost in higher lineages or adapted for new functionality.

Although we found no evidence for any broadly conserved sub-families, there was a single SR sub-family shared between members of the Viridiplantae, a red alga and the bilateral metazoans: SC35 (SRSF2). The sharing of this sub-family across so many diverse organisms might be due to its function not only in splicing (5′ and 3′ splice site recognition and interacting with U170-K and U2AF35) [35] but also because of its facilitation of transcription elongation of nascent transcripts [57]. Presumably, this integration of transcription and splicing could very well be a fundamental biological process that has been conserved throughout multiple eukaryotic lineages.

Furthermore, our results support the idea that there are three plant-specific families: RS, RS2Z and SCL. Previous studies have often been limited in their phylogenetic scope, that is, often only a small subset of organisms and their SR gene repertoires were studied, such as human, drosophila, roundworm, fission yeast, moss, rice and Arabidopsis [24], [44]. By including multiple species from divergent lineages, we were able to categorize SR genes into sub-families that will not only help in answering questions related to lineage-specific sub-family expansion (see below) but also enable experimental design for gene knockout studies.

### SR sub-family expansion in plants and selective pressures

Based on work in Arabidopsis and rice, it was assumed that plants have the largest inventory of SR genes of any eukaryotes [36]. The work presented here, with the inclusion of 27 different eukaryotic organisms, confirms this general trend (Figure 2). The flowering plants (Arabidopsis, poplar, rice, soybean, sorghum and maize) have double or nearly double the number of SR genes found in verterbrates (Figure 2). However, *V. vinifera* has the fewest SR genes of all the higher plants (Magnoliophyta). If one considers the influence of whole genome duplication events in the histories of flowering plants, this reduced number of SR genes in *V. vinifera* makes sense, since this genome has not undergone a recent duplication event, and instead experienced a paleo-hexaploidization event after the divergence from the monocots but before the separation of the Eurosids [58].

The large number of SR genes in flowering plants can be attributed to whole genome duplication events, as previously mentioned. As whole genome duplication appears to be the rule rather than the exception within flowering plants, it is not surprising that these organisms would have a larger inventory of SR genes than the vertebrates. *Glycine max*, which has the most SRs of any organism we studied, is estimated to have undergone two duplication events, estimated at 59 and 13 million years ago [59]. Even the moss, *P. patens* is estimated to have a recent genome duplication in its past, occurring between 30–60 million years ago [62], around the same time that Arabidopsis experienced its most recent duplication event [60].

While there are many SR genes in plants, what remains to be understood is why there is a need for so many splicing regulators. Given our analysis using microarray expression data for Arabidopsis, rice, soybean and maize, it appears that expression levels between the members of a duplicate pair are tightly regulated, with very few instances of overlapping expression magnitudes within the same developmental stages (Figure 4). The overwhelming majority of SR homologs experiencing purifying selection points to a post-duplication scenario of maintaining SR gene structure, form and function, albeit while reducing genetic redundancy via regulated and divergent gene expression. Such a situation might arise in evolution when there is a need for genetic robustness against potential null mutations [61]. However, an interesting case for novel function over redundancy is visible with respect to the SC35a gene in maize. ZmSC35a was one of the six genes with evidence to suggest that it is evolving under positive selection (see above and Figure S8). Considering its expression profile against that of its paralog (last panel in Figure 4), it clearly overlaps in expression magnitude across 57 different arrays with ZmSC35b during the developmental stage of stem elongation. While most of the pairs may be experiencing purifying selection and may have redundant or sub-functions, ZmSC35a may be one of the salient examples of a duplicated gene taking on novel function.

The six genes with *K_a_*/*K_s_*>0.9 (see above and Figure S8) all belong to the lineage of monocots in the SR and SC sub-families. This implies that these genes are undergoing positive selection in the monocot lineage or have been accumulating non-synonymous mutations in the ancestral population predating the emergence of the monocots. The second argument is more likely to be true because it does not require the assumption that independent positive selection on the same gene occurs in all three monocot species.

Additionally, our analysis of RNA binding motifs within the plant-enriched sub-families is further indication that many residues within SR proteins are highly conserved and under purifying selection. However, if many of the residues within RRM regions in a sub-family are conserved, how might binding specificity be achieved among sub-family members from a single species? First, for each sub-family, there are multiple RNA binding motifs (underlined regions in Figure 3). Although many residues may be conserved within a sub-family in a particular binding region, certain residues between binding regions are also conserved. However, in every predicted binding region there are at least three highly variable positions bordered by highly constant positions (except for the third binding motif in SC35, *sRGFAFVR*). Nevertheless, conserved and variable residues within binding regions are only partial players in RNA binding specificity. Other factors may influence specific binding or even be required to activate binding, such as phosphorylation of RS domains [62], even if RS domains may be interchangeable [63].

### Alternative splicing of SR genes is a common characteristic among eukaryotes

The SR gene family comprises important regulators of both constitutive and alternative splicing and are extensively alternatively spliced themselves [34]. Thus far, the investigation of AS of SR genes has been limited to a subset of model organisms, particularly mouse and human [64], drosophila [65], roundworm [66], Arabidopsis and rice [36]. Though AS of SR genes has been shown to be a common occurrence in these organisms, what has not been addressed is whether AS of SR genes is a common eukaryotic trait. Consolidation of information for 27 organisms and their SR gene repertoires allows perspective into the extent of AS across organisms, the preferred types of AS events and how these events can vary by organism or specific SR sub-family.

We observed AS in SR genes across 20 organisms with sufficient EST/cDNA data (see Table 2). Mouse and human were the only two organisms to have AS events in each of their SR genes. No AS was found in three organisms (*D. discoideum*, *P. falciparum* and *P. sojae*), which are considered as “basal” eukaryotes, with a highly reduced number of SR genes in their genomes relative to the remaining 20 organisms (see Figure 2). Their reduced number of SR genes is most likely indicative of their genomes having a relatively low number of introns [67], and the lack of AS found in *D. discoideum*, *P. falciparum* and *P. sojae* SR genes further supports this idea.

Recent work in Arabidopsis [43] and human and mouse [64] has suggested that regulated unproductive splicing is a prominent means for controlling functional SR transcript abundance. The overwhelming occurrence of AS in our sampled SR genes (Table 2, Figure 6 and Figure S10) is highly suggestive of AS having a critical role in the regulation of functional SR transcript abundance across multiple eukaryotic lineages. An interesting peculiarity is evident when considering that the Viridiplantae generally have the largest number of SR genes relative to the Euteleostomi: a larger number of SR genes does not necessarily translate into a higher number of genes subjected to AS. As was mentioned in the introduction, recent studies revealed that 95–100% of all human multi-exon genes undergo AS [12], [13], whereas roughly only 40% of multi-exon genes experience AS in plants [14]–[17]. However, whether or not a massive increase in expression data for plants will augment these percentages remains to be seen.

### Differences in AS event types

Performing a large comparative analysis of SR genes across species allows us to discern which alternative splicing event types are predominant. Across the 20 organisms we sampled, alternative 3′ splicing is the most common AS event type among SR genes (134 genes), followed by intron retention (111 genes), alternative 5′ splicing (109 genes), skipped exons (106 genes) and finally alternative 3′ and 5′ events (29 genes). As we saw earlier, intron retention was not the universally abundant AS event type in the Viridiplantae and was only the most prevalent AS type in two of the nine photosynthetic eukaryotes (normalized averages in Figure S10). This suggests that different plant species might have specific preferences towards generating alternative splice forms of their SR genes or that the varying proportions of AS event types in Figure S10 is the result of variation in EST/cDNA tissue sources. In contrast to the Viridiplantae, the Euteleostomi generally display a preference for exon skipping over intron retention, which agrees with previous genome-wide studies of alternative splicing in metazoans [18]. Interestingly, different SR sub-families show different levels of AS and preferences for AS event types. In general, there is a higher incidence of alternatively spliced SR genes in plant-enriched sub-families as well as a higher number of IR and Alt 3′ events per SR gene (green shaded boxes in Figure S11), whereas there is a lower number of alternatively spliced SR genes in non-photosynthetic sub-families and a lower incidence of IR events (blue shaded boxes in Figure S11). These results suggest that specific sub-families rely on different types of AS to either generate novel protein forms with altered RRM binding domains [42], altered RS domains which may have implications on nuclear localization of the SR protein [24], or to affect the number of transcripts subjected to nonsense mediated decay [64].

### Conclusions

We performed a large-scale comparative analysis of one of the most critical gene families involved in a fundamental biological process across multiple eukaryotic lineages. The SR gene family can be split into five major groups, which can be further separated into at least 11 sub-families. Based on these groupings, we applied a standardized nomenclature to plant SR genes that will be helpful for future studies. Most flowering plants possess double or nearly double the number of SR genes than vertebrates presumably due to extensive ancestral genome duplications. Furthermore, the majority of SR genes in flowering plants experience purifying selection and one member of a gene pair (in Arabidopsis, rice, soybean and maize) is preferentially expressed over the other throughout plant development. SR genes are conserved in sequence and domain organization yet differ in number and sub-family distribution across lineages and experience different preferences in alternative splicing. The work here has implications on the general evolution of homologous genes, for biological experimentation and differential regulation of SR gene expression by different types of alternative splicing.

## Methods

### Species selection

We employed several criteria to determine which organisms would be sampled in our study. These included: completeness and availability of genomic sequence, availability and bulk of cDNA or EST data and phylogenetic diversity. The major element influencing the selection of species was that of EST information, since this was the limiting factor. We used NCBI's dbEST [68] in order to glean information on the abundance of available transcripts per organism contained within the NCBI genome databases. Based on the EST counts per organism and their phylogenetic diversity, 27 species were selected and included in the alternative splicing analysis. Details of the procedure are described below.

### Organism sampling and SR sequence acquisition

To begin the assessment of the genomic inventory of SR genes in eukaryotes, we selected taxa based on completeness of genome sequencing efforts and their phylogenetic diversity inferred from NCBIs taxonomy browser (http://www.ncbi.nlm.nih.gov/Taxonomy/CommonTree/wwwcmt.cgi; Figure S1). We sampled a total of 27 organisms with fully sequenced genomes that ranged from plants, animals and fungi (Opisthokonts) to Amoebozoa, Stramenopiles and the Alveolata [69]. Once the organisms were chosen, SR amino acid sequences were obtained through either literature searches (*Homo sapiens* [64], *Caenorhabditis elegans* [66], *Drosophila melanogaster* [65], *Schizosaccharomyces pombe* [37], *Arabidopsis thaliana* [70] and *Oryza sativa* [44]) or via hidden markov model (HMM) searches using HMMER3 [71] (see Table 1) of downloaded protein databases.

We used a combination of HMM [71] and BLASTP [72] searches to find and then verify that putative sequences were SR gene homologs. We constructed three separate HMMs: one for the Viridiplantae (vHMM), one for the Fungi/Metazoa (fmHMM) and one for the Amoebozoa, Stramenopiles and Alveolata (asaHMM). The vHMM was composed of globally aligned [73] SR proteins of *Arabidopsis thaliana*, *Oryza sativa*, preliminary BLASTP candidate sequences from *Populus trichocarpa* and *Chlamydomonas reinhardtii*. Using this vHMM, we then searched downloaded protein databases of *Glycine max*, *Vitis vinifera*, *Zea mays*, *Sorghum bicolor*, *Selaginella moellendorfii*, *Physcomitrella patens*, *Chlorella vulgaris* and *Cyanidioschyzon merolae* (database references in Table 1). After re-searching downloaded databases of *Chalmydomonas reinhardtii* and *Populus trichocarpa* with this HMM, we then used the full sequence E-value from the HMMER3 output to exclude hits with an E-value greater than 10^−3^ to generate a set of candidate SR proteins. Next, we blasted each of the candidate SR proteins against the nr protein database at NCBI to validate which of the candidate sequences could be further excluded based on sequence similarity to known non-SR proteins. All remaining candidates were then manually examined for the occurrence of a one or two N-terminal RRMs and a C-terminal SR domain with at least three SR dipeptides. These were then submitted to Interproscan for domain searches to elucidate positions of their RRMs [74], [75].

A similar process was performed with the fmHMM and the asaHMM. The only differences being the underlying sequences used in the construction of the respective HMMs. The fmHMM was composed of known SRs from *Homo sapiens*, *Caenorhabditis elegans*, *Drosophila melanogaster* and *Schizosaccharomyces pombe*, whereas the asaHMM was comprised of SRs from *Homo sapiens*, *Ciona intestinalis*, *Drosophila melanogaster*, *Neurospora crassa*, *Arabidopsis thaliana* and *Chlamydomonas reinhardtii*. Using the fmHMM, we searched downloaded protein sequence databases of *Mus musculus*, *Gallus gallus*, *Xenpus tropicalis*, *Danio rerio*, *Branchiostoma floridae*, *Ciona intestinalis*, *Anopheles gambiae*, *Aedes aegypti* and *Neurospora crassa* (references in Table 1). The asaHMM was used to search downloaded databases of *Plasmodium falciparum*, *Phytopthora sojae* and *Dictyostelium discoidum*. As with the vHMM search process, the same data filtering steps were taken to derive putative SR gene homologs within the Fungi/Metazoa and other eukaryotes.

The following sequences that did not begin with methionine were removed: Chlv31017 (*Chlorella vulgaris*), Sb0514s002010 and Sb09g004685 (*Sorghum bicolor*), and Smo36388 (*Selaginella moellendorfii*). All accession numbers for all SR proteins used in these analyses are available in Table S2.

### Alignment procedure

The resulting 272 SR proteins from the searches described above were initially aligned using DIALIGN-TX [76], [77] with default parameters. The RNA recognition motifs (RRMs) were extracted from the full-length amino acid sequences of the SR proteins based on their SMART [78] prediction coordinates from Interproscan searches [74], [75]. A preliminary UPGMA tree was constructed to evaluate the aligned RRMs. There were no instances of a crisscrossed matchup of an N-terminal RRM with a C-terminal RRM.

After the above determinations, all N-terminal RRMs were aligned separately from those sequences harboring a C-terminal RRM, which were also aligned separately. Here, we used FSA [79] for the alignment of the RRMs because of its explicit consideration of insertions that should not align, which would otherwise confound our gene tree analyses by over-estimating the substitution rates. The disjoint alignments of sequences with two RRMs were then concatenated and any columns that would be considered gap-only if a single sequence did not cause an unalignable insertion to exist were removed. The amino acid sequence of the RRM in seventeen taxa was identical. Of these, one representative RRM was selected for use in gene tree construction, reducing the data matrix to 255 taxa. Twenty-eight columns of the 353 total characters in the alignment were constant, 267 were parsimony-informative and 58 were uninformative variable characters. The RRM alignment used in this analysis is available in fasta format in Dataset S1.

### Gene tree inferences

The alignment constructed as described above was input into PROTTEST version 2.4 [80] and assessed for the best fitting model of amino acid substitution. The best scoring model with the fewest number of parameters was the LG model with a gamma shape distribution for rate heterogeneity (LG+G, lnl: −24515.47). Next, two maximum likelihood (ML) methods and a parsimony method were used to construct gene trees of the 255 SR proteins. We used the parallel threads implementation of RAxML version 7.2.6 [81], [82] to perform 2000 rapid bootstraps and search for the best known tree under the LG+G model (lnl: −23016.56). We used Garli version 1.0 as the second ML tree search method to conduct ML analyses on another 1000 bootstrap replicates [83]. One thousand parsimony bootstrap replicate searches were conducted in Phylip version 3.69 using the protpars program and randomized input order of sequences (10 jumbles) [84]. Bootstrap support values from all three analyses were then mapped onto the best scoring ML tree from the RAxML analysis.

### Genomic and cDNA/EST sequences for Alternative Splicing (AS) analysis

In addition to acquiring amino acid sequences of the SR genes, we also obtained full-length genomic sequences from the corresponding databases in Table 1. Next, we performed a series of MEGABLAST searches against NCBI's dbEST using each of the genomic sequences for each of the organisms in order to collect EST data to be used in the analysis of alternative splicing (AS) for the organisms under study. MEGABLAST searches were also conducted against the nr nucleotide database to acquire any full-length cDNAs that were available.

### Alternative splicing analysis

Of the 27 eukaryotic organisms sampled in this study, 24 had EST data obtained from the MEGABLAST searches described above, except for *Selaginella moellendorfii*, *Chlorella vulgaris* and *Cyanidioschyzon merolae*. The genomic sequences and transcript sequences were then fed into an in-house generated pipeline to assess the extent of AS among the SR genes in these 24 organisms.

We used a modified version of the Sircah program [85] to detect possible AS events from a set of aligned transcripts as described in [46]. To provide meaningful counts for alternative splicing events, we established rules for each event type. For our analysis we counted the number of events supported by EST transcripts (see Figure S13).

### Normalization of Alternative splicing measurements

To compare alternative splicing evidence between SR genes from different organisms, we applied an approach similar to that used in [18], [86]. We ran 100 trials in which we randomly selected a fixed number of 15 ESTs for each SR gene in each organism. Genes that had fewer than the required 15 EST alignments were omitted from our analysis. We selected a threshold of 15 ESTs to provide enough sensitivity to illuminate differences between species while permitting analysis on all but the three poorly represented species. We ran a modified version of Sircah [46], [85] on the randomly selected ESTs to generate statistics on the number of alternative splicing events. In each trial and for each organism we counted the number of genes used in the trial, the number of genes that exhibited alternative splicing and the number of alternative splicing events: intron retention, skipped exon, alternative 5′ site, alternative 3′ site and simultaneous 3′/5′ (Alt B).

## Supporting Information

Figure S1 **Phylogeny of the 27 sampled organisms.** Phylogeny was determined using the NCBI taxonomy browser (http://www.ncbi.nlm.nih.gov/Taxonomy/CommonTree/wwwcmt.cgi). Although the NCBI taxonomy browser is not an authoritative source for phylogenetics, for the purposes of illustrating the diversity inherent to the organisms sampled in this study, it readily describes the broad evolutionary relationships among them.
(TIFF)

Figure S2 **Full Cladogram.** Uninterrupted cladogram, with sub-families annotated with labels and colors. Plotted onto the branches are bootstrap support values from RAxML (top left), GARLI (top right) and maximum parsimony (bottom). The “-” symbols denote a lack of support for a particular grouping, which were typically from the parsimony analysis. If a sequence is followed by equality, it represents one or more other sequences that had exactly identical RRM(s) in the multiple alignment and were not included in the gene tree inference. Red branches indicate branch lengths greater than 0.75. The *P. paten*s sequence is underlined because it contains a Zinc knuckle, whereas the remaining sequences do not (see text).
(TIFF)

Figure S3 **Expansion of SCL, SC35 (SRSF2) and SRp38 (SRSF10) sub-families.** The SCL and photosynthetic members of SC35 are shown in green, SRp38 (SRSF10) members are shown in blue. Plotted onto the branches are bootstrap support values from RAxML (top left), GARLI (top right) and maximum parsimony (bottom). The “-” symbols denote a lack of support for a particular grouping, which were typically from the parsimony analysis. If a sequence is followed by equality, it represents one or more other sequences that had exactly identical RRM(s) in the multiple alignment and were not included in the gene tree inference. Red branches indicate branch lengths greater than 0.75. The *P. patens* sequence is underlined because it contains a Zinc knuckle, whereas the remaining sequences do not (see main text). Taxon labels use the same species prefixes as described in Figure 1 of the main text.
(TIFF)

Figure S4 **Expansion of RS and SRp54 (SRSF11) sub-families.** RS (green) and SRp54 (blue) are shown in expanded form. Labeling conventions are as described in previous figures.
(TIFF)

Figure S5 **Expansion of SRp40 (SRSF10), SRp55/75 (SRSF6/SRSF4) and RS2Z sub-families.** SRp55/75 (SRSF6/SRSF4) (top blue clade) and SRp40 (SRSF5) (middle and bottom blue clades) are shown in expanded form. The RS2Z plant-specific sub-family is shown in expanded form. A *G. max* sequence is underlined because it does not possess the canonical double Zinc knuckle domains characteristic of this sub-family (see text). Labeling conventions are as described in previous figures.
(TIFF)

Figure S6 **Expansion of SR and SF2 (SRSF1) sub-families.** SR (green) and SF2 (blue) clades are shown in expanded form. Labeling conventions are as previously described.
(TIFF)

Figure S7 **Expansion of RSZ and 9G8/SRp20 (SRSF7/SRSF3) sub-families.** RSZ (green) and 9G8/SRp20 (SRSF7/SRSF3) (blue) are shown in expanded form. The two algal species are underlined because they do not possess the canonical Zinc knuckle domain that characterizes this sub-family. Labeling conventions are as described in previous figures.
(TIFF)

Figure S8 **Orthologous pairwise** ***K*** **_a_/** ***K*** **_s_ ratios for plant sub-families.** Pairwise comparisons of orthologous SR genes are shown. Ratios less than or equal to 0.1 are indicated by blue crosses, ratios in between 0.1 and 0.9 are shown as yellow crosses and ratios greater than or equal to 0.9 are depicted as red crosses.
(TIFF)

Figure S9 **Expression of non-paralogous Arabidopsis SR genes.** Gene expression data for various developmental stages were taken from the Genevestigator database [54] and plotted for each SR gene that does not have a paralog. The numbers below the x-axis indicate the number of microarray experiments that underlie the average intensity value plotted on the y-axis.
(TIFF)

Figure S10 **AS event type prevalence by organism.** Based on the normalization procedure described in the methods, five different AS event types were counted (IR, intron retention; SE, skipped exon; Alt 3′, alternative 3′; Alt 5′, alternative 5′ and Alt B, both Alt 3′ and Alt 5′ of the same intron). The y-axis shows the mean AS event type per gene experiencing AS in the normalization procedure. The arrangement of the shaded panels and numbers below the taxon names are similar to what is depicted in Figure 6.
(TIFF)

Figure S11 **Family-wise AS comparisons.** Panel A depicts the normalized proportion of genes undergoing AS per sub-family by averaging the values across the 100 trials in triplicate. Shading conventions are as previously described. Panel B shows the mean AS event type per gene experiencing AS in the normalization procedure but according to sub-family rather than organism (c.f. Figure S10). The Viridiplantae sub-families are shaded in green whereas the others are shaded in blue. The numbers below the sub-families designate the number of genes with AS in that particular sub-family. SRp40 and SRp55/75 are separated here to highlight differences between vertebrates and insects.
(TIFF)

Figure S12 **Log Median ESTs/cDNAs per organism.** The median number of ESTs/cDNAs per gene per organism is presented on a log scale, with raw values indicated within the bars.
(TIFF)

Figure S13 **How AS event types are counted.** As a simple example, consider the transcripts given in Panel A. Although there are two retained introns, the transcripts support only one intron retention event in which both introns are retained simultaneously. Consequently, for this graph we count a single intron retention event. A more complicated example is shown in Panel B. The graph has two retained introns for which three combinations are supported by EST transcripts. Additionally, there are two alternate 5′ events supported by transcripts and an alternate 3′ event. In this case, we count three intron retention events, two alternate 5′ events and a single alternate 3′ event. The rules for cassette exons are analogous to those for intron retention: when there is evidence of multiple skipped exons in a gene, we count number of distinct EST transcripts that support each combination. For alternative 3′ and 5′ splice sites, we use the most prevalent splice site (the one supported by a plurality of EST transcripts) and simply count the number of alternatives. When we cannot determine a prevalent form, we use the splice site that yields the longest intron. We distinguish between alternate 3′ sites (Alt 3′), alternate 5′ sites (Alt 5′) and simultaneous 3′/5′ events (Alt B). We count Alt B events whenever an alternative 5′ site is paired with the same alternate 3′ site in all transcripts. For example, in Panel C the alternate 3′ and 5′ splice sites are paired, so this will be counted as a single Alt B event. We incorporated our counting rules into our modified version of Sircah and generated statistics for each kind of AS event.
(TIFF)

Table S1 **Table of the distribution of ESTs/cDNAs per organism.** Table showing the count of SR genes in each organism that have the required number of ESTs, where the number of required ESTs ranges from 2 to 20.
(PDF)

Table S2 **Information on SR genes used in this study.** Excel file with accession numbers, sub-family designations, protein lengths, intron number, strand information, molecular weights, revised nomenclature and domain locations and organization of all SR genes used in this study.
(XLSX)

Dataset S1 **Fasta alignment file.** Plain text file of the RRM alignment used to infer the gene trees in this study.
(TXT)
