# Supplementary figures and images for: Comparative Analysis of Serine/Arginine-Rich Proteins across 27 Eukaryotes: Insights into Sub-Family Classification and Extent of Alternative Splicing
Source: PLoS One. 2011 Sep 14;6(9):e24542. doi: 10.1371/journal.pone.0024542 (PMC3173450; doi:10.1371/journal.pone.0024542)

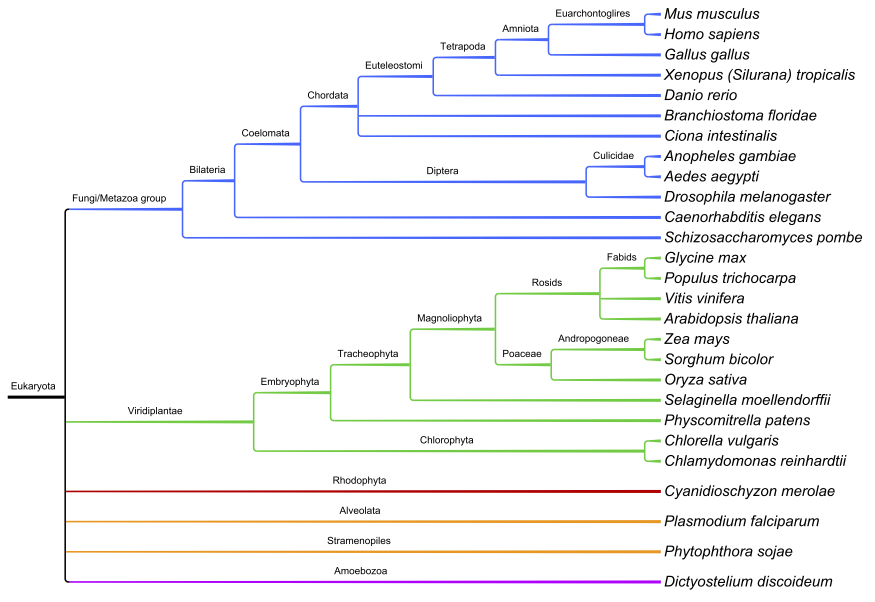

Supplement: Figure S1 — Phylogeny of the 27 sampled organisms. Phylogeny was determined using the NCBI taxonomy browser (http://www.ncbi.nlm.nih.gov/Taxonomy/CommonTree/wwwcmt.cgi). Although the NCBI taxonomy browser is not an authoritative source for phylogenetics, for the purposes of illustrating the diversity inherent to the organisms sampled in this study, it readily describes the broad evolutionary relationships among them. (TIFF) [file pone.0024542.s001.tiff]

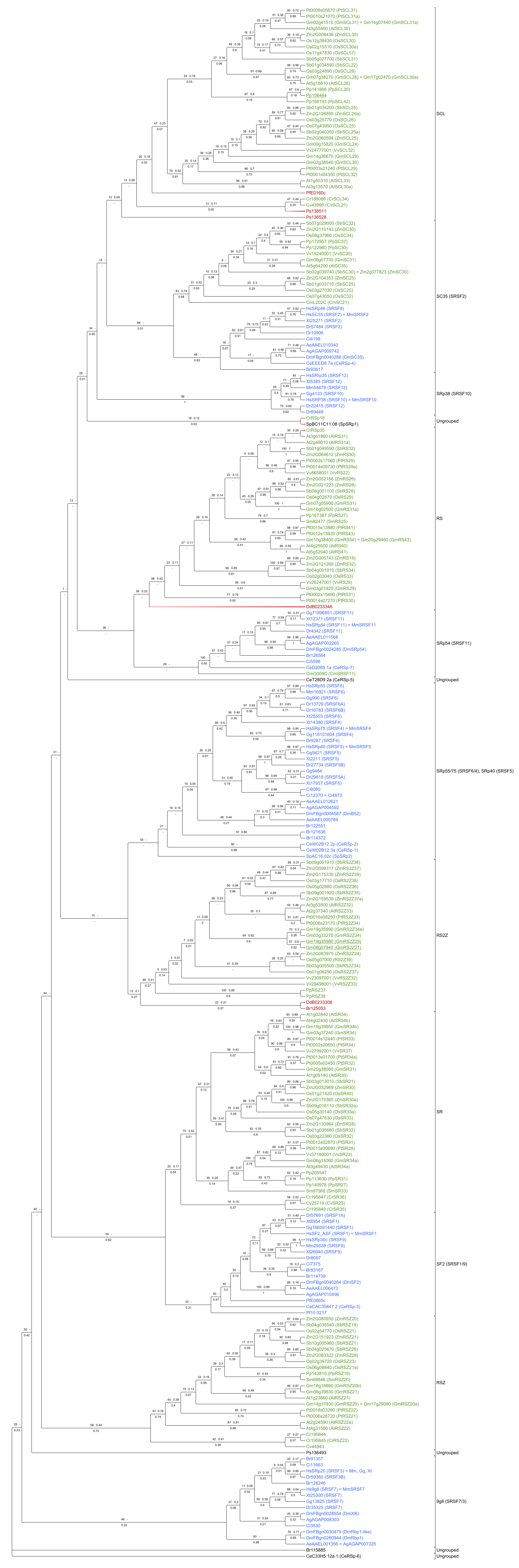

Supplement: Figure S2 — Full Cladogram. Uninterrupted cladogram, with sub-families annotated with labels and colors. Plotted onto the branches are bootstrap support values from RAxML (top left), GARLI (top right) and maximum parsimony (bottom). The “-” symbols denote a lack of support for a particular grouping, which were typically from the parsimony analysis. If a sequence is followed by equality, it represents one or more other sequences that had exactly identical RRM(s) in the multiple alignment and were not included in the gene tree inference. Red branches indicate branch lengths greater than 0.75. The P. patens sequence is underlined because it contains a Zinc knuckle, whereas the remaining sequences do not (see text). (TIFF) [file pone.0024542.s002.tiff]

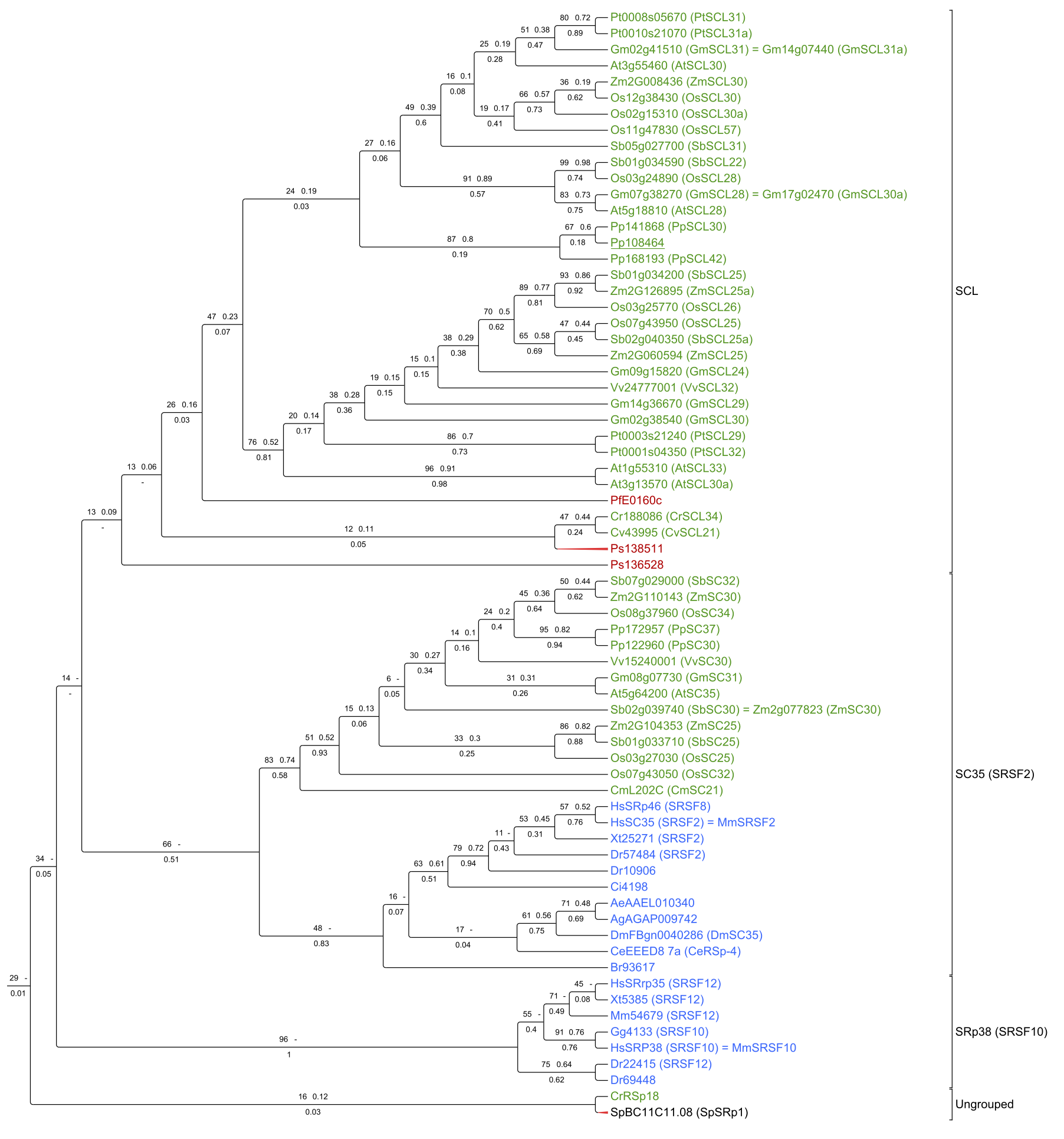

Supplement: Figure S3 — Expansion of SCL, SC35 (SRSF2) and SRp38 (SRSF10) sub-families. The SCL and photosynthetic members of SC35 are shown in green, SRp38 (SRSF10) members are shown in blue. Plotted onto the branches are bootstrap support values from RAxML (top left), GARLI (top right) and maximum parsimony (bottom). The “-” symbols denote a lack of support for a particular grouping, which were typically from the parsimony analysis. If a sequence is followed by equality, it represents one or more other sequences that had exactly identical RRM(s) in the multiple alignment and were not included in the gene tree inference. Red branches indicate branch lengths greater than 0.75. The P. patens sequence is underlined because it contains a Zinc knuckle, whereas the remaining sequences do not (see main text). Taxon labels use the same species prefixes as described in Figure 1 of the main text. (TIFF) [file pone.0024542.s003.tiff]

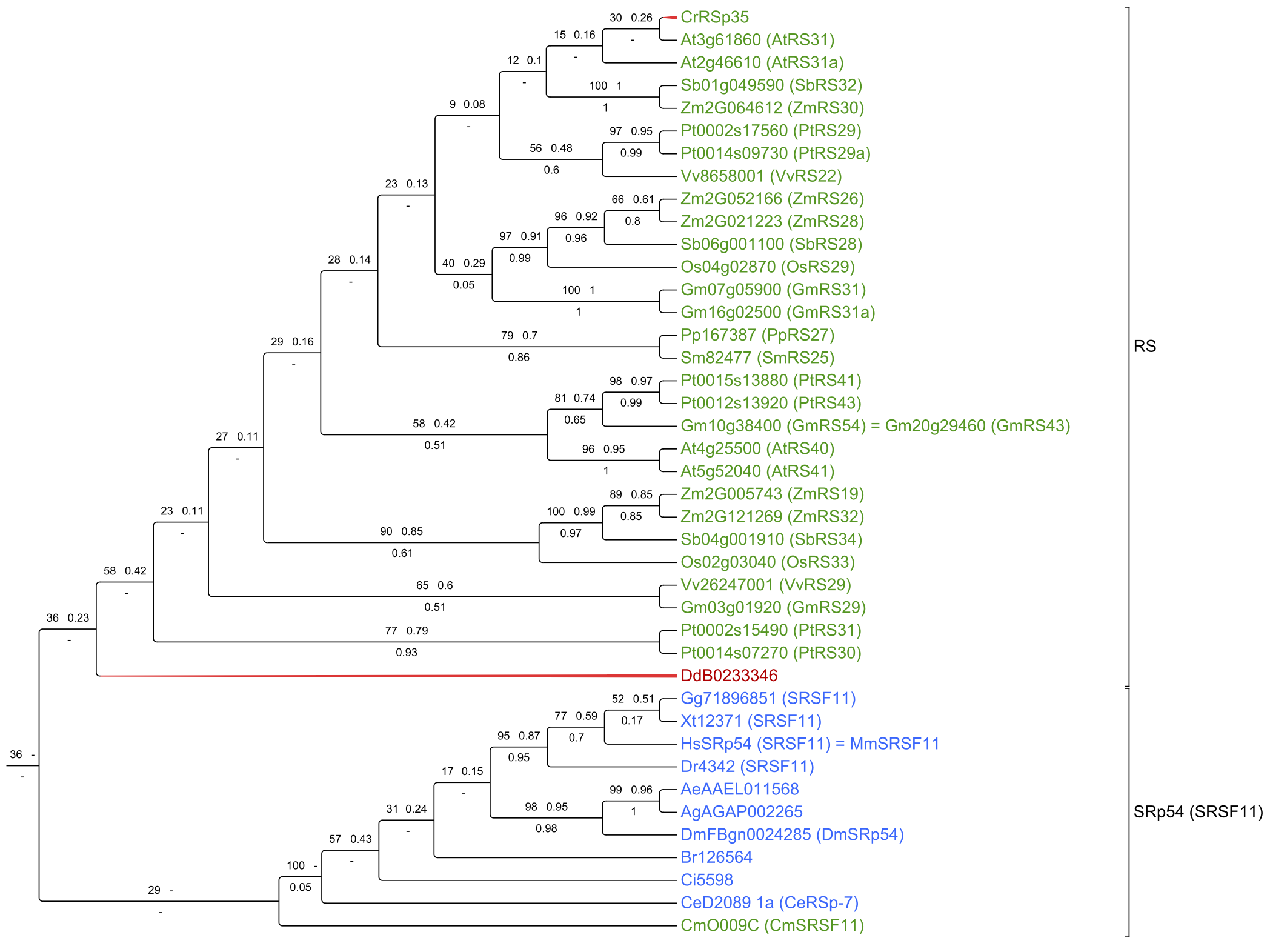

Supplement: Figure S4 — Expansion of RS and SRp54 (SRSF11) sub-families. RS (green) and SRp54 (blue) are shown in expanded form. Labeling conventions are as described in previous figures. (TIFF) [file pone.0024542.s004.tiff]

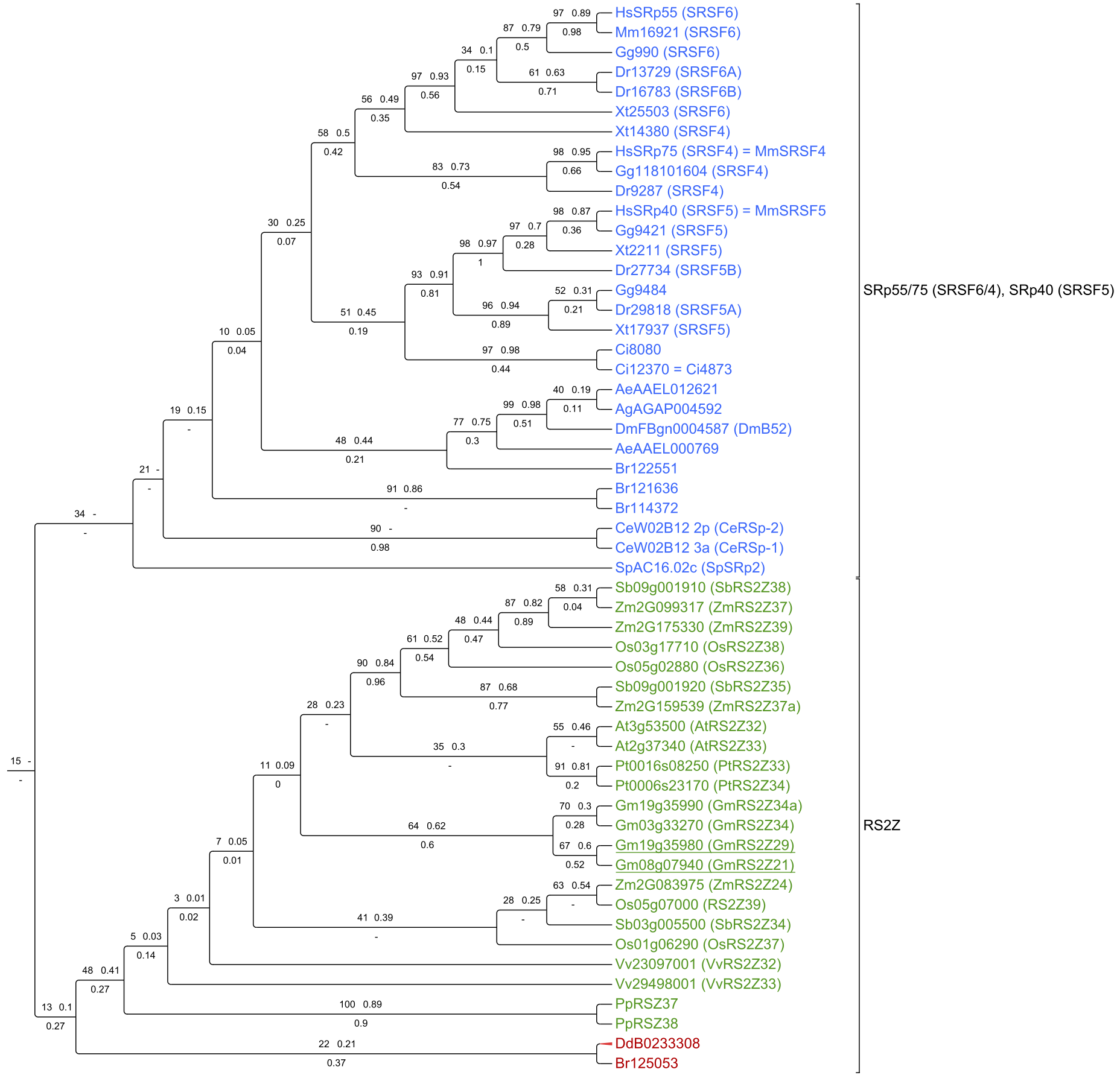

Supplement: Figure S5 — Expansion of SRp40 (SRSF10), SRp55/75 (SRSF6/SRSF4) and RS2Z sub-families. SRp55/75 (SRSF6/SRSF4) (top blue clade) and SRp40 (SRSF5) (middle and bottom blue clades) are shown in expanded form. The RS2Z plant-specific sub-family is shown in expanded form. A G. max sequence is underlined because it does not possess the canonical double Zinc knuckle domains characteristic of this sub-family (see text). Labeling conventions are as described in previous figures. (TIFF) [file pone.0024542.s005.tiff]

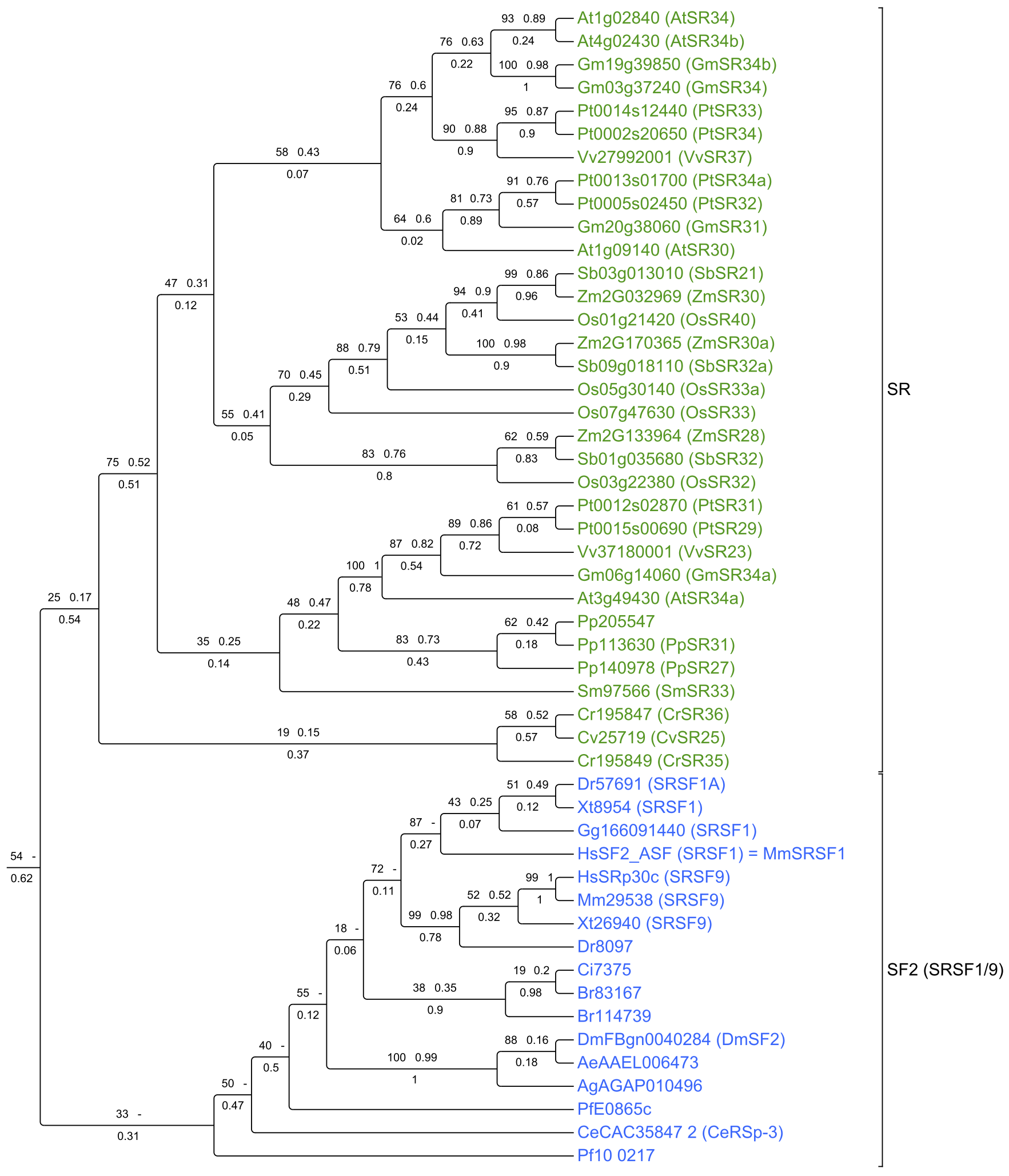

Supplement: Figure S6 — Expansion of SR and SF2 (SRSF1) sub-families. SR (green) and SF2 (blue) clades are shown in expanded form. Labeling conventions are as previously described. (TIFF) [file pone.0024542.s006.tiff]

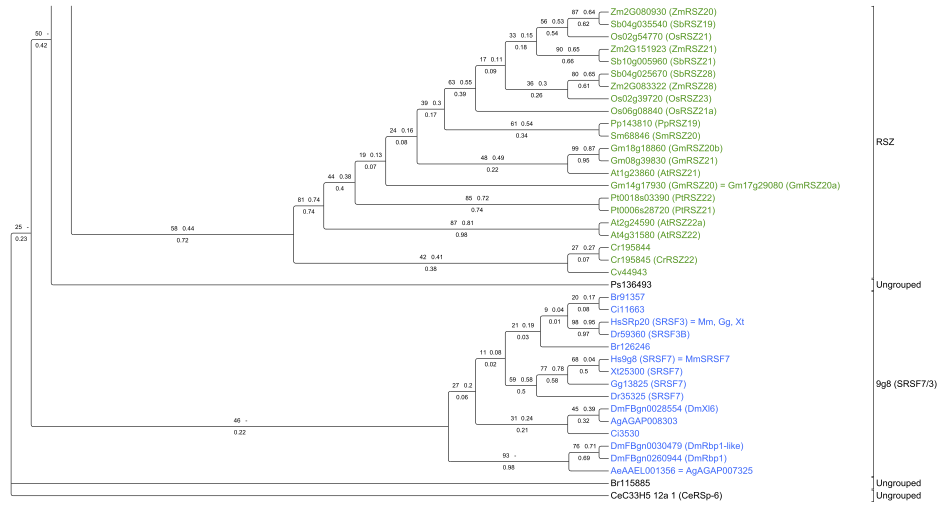

Supplement: Figure S7 — Expansion of RSZ and 9G8/SRp20 (SRSF7/SRSF3) sub-families. RSZ (green) and 9G8/SRp20 (SRSF7/SRSF3) (blue) are shown in expanded form. The two algal species are underlined because they do not possess the canonical Zinc knuckle domain that characterizes this sub-family. Labeling conventions are as described in previous figures. (TIFF) [file pone.0024542.s007.tiff]

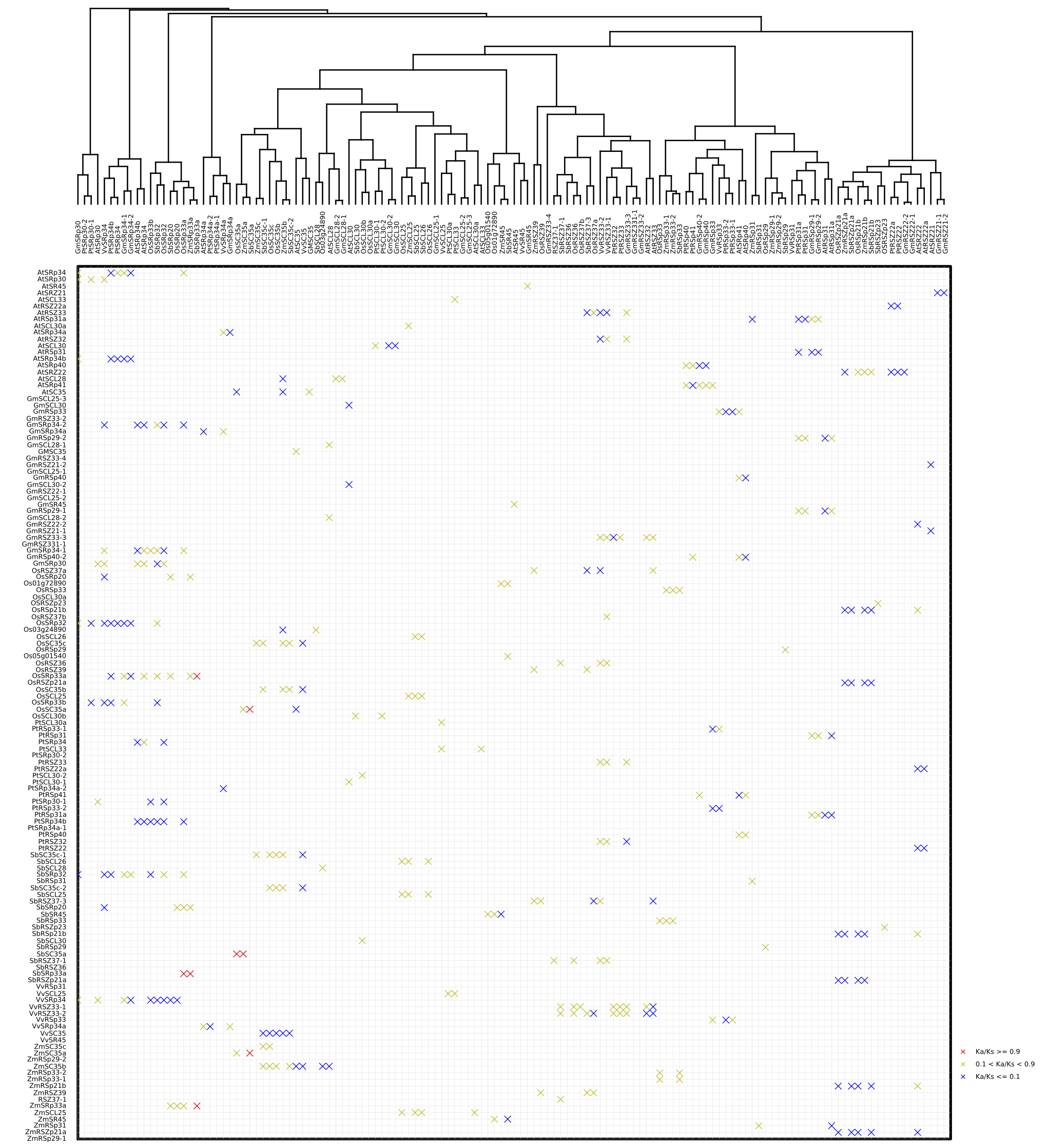

Supplement: Figure S8 — Orthologous pairwise K a/ K s ratios for plant sub-families. Pairwise comparisons of orthologous SR genes are shown. Ratios less than or equal to 0.1 are indicated by blue crosses, ratios in between 0.1 and 0.9 are shown as yellow crosses and ratios greater than or equal to 0.9 are depicted as red crosses. (TIFF) [file pone.0024542.s008.tiff]

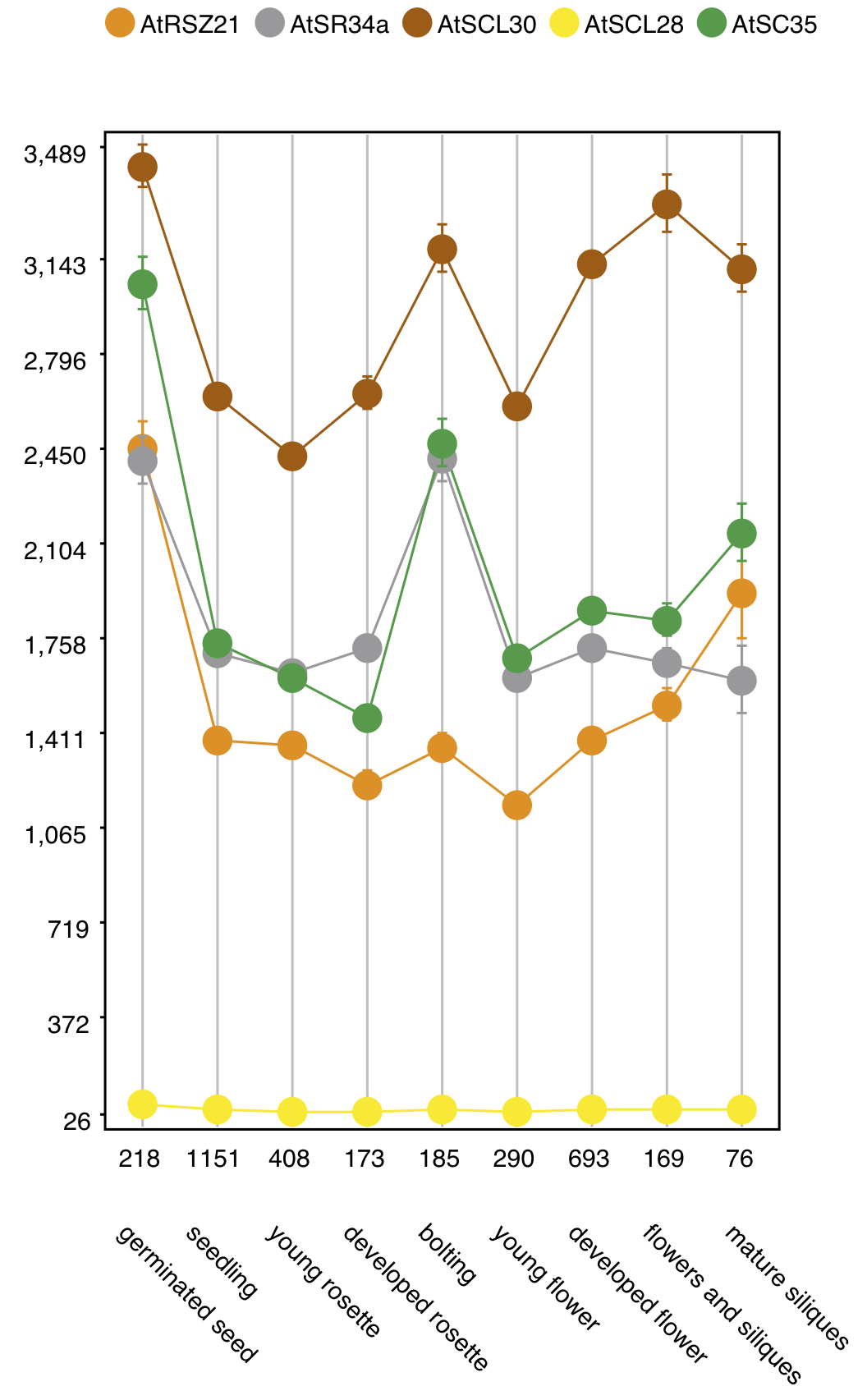

Supplement: Figure S9 — Expression of non-paralogous Arabidopsis SR genes. Gene expression data for various developmental stages were taken from the Genevestigator database [54] and plotted for each SR gene that does not have a paralog. The numbers below the x-axis indicate the number of microarray experiments that underlie the average intensity value plotted on the y-axis. (TIFF) [file pone.0024542.s009.tiff]

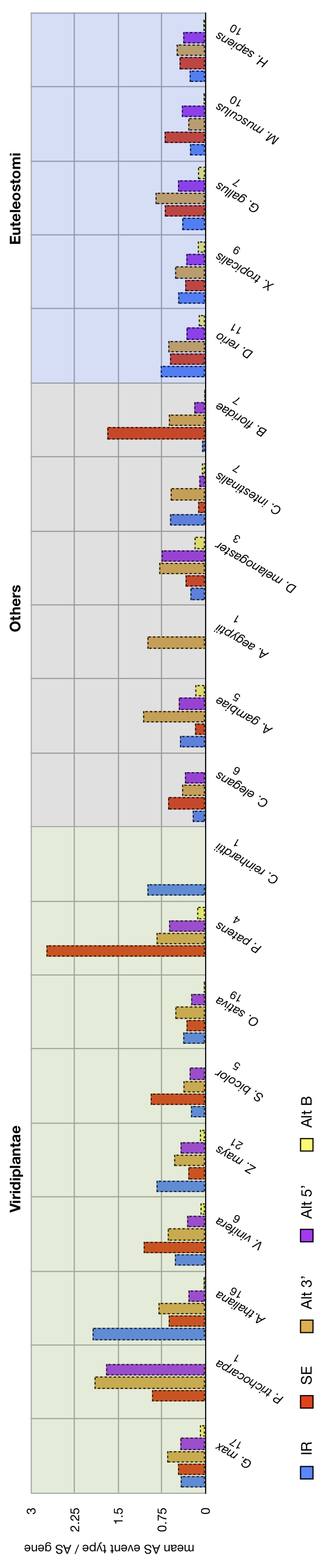

Supplement: Figure S10 — AS event type prevalence by organism. Based on the normalization procedure described in the methods, five different AS event types were counted (IR, intron retention; SE, skipped exon; Alt 3′, alternative 3′; Alt 5′, alternative 5′ and Alt B, both Alt 3′ and Alt 5′ of the same intron). The y-axis shows the mean AS event type per gene experiencing AS in the normalization procedure. The arrangement of the shaded panels and numbers below the taxon names are similar to what is depicted in Figure 6. (TIFF) [file pone.0024542.s010.tiff]

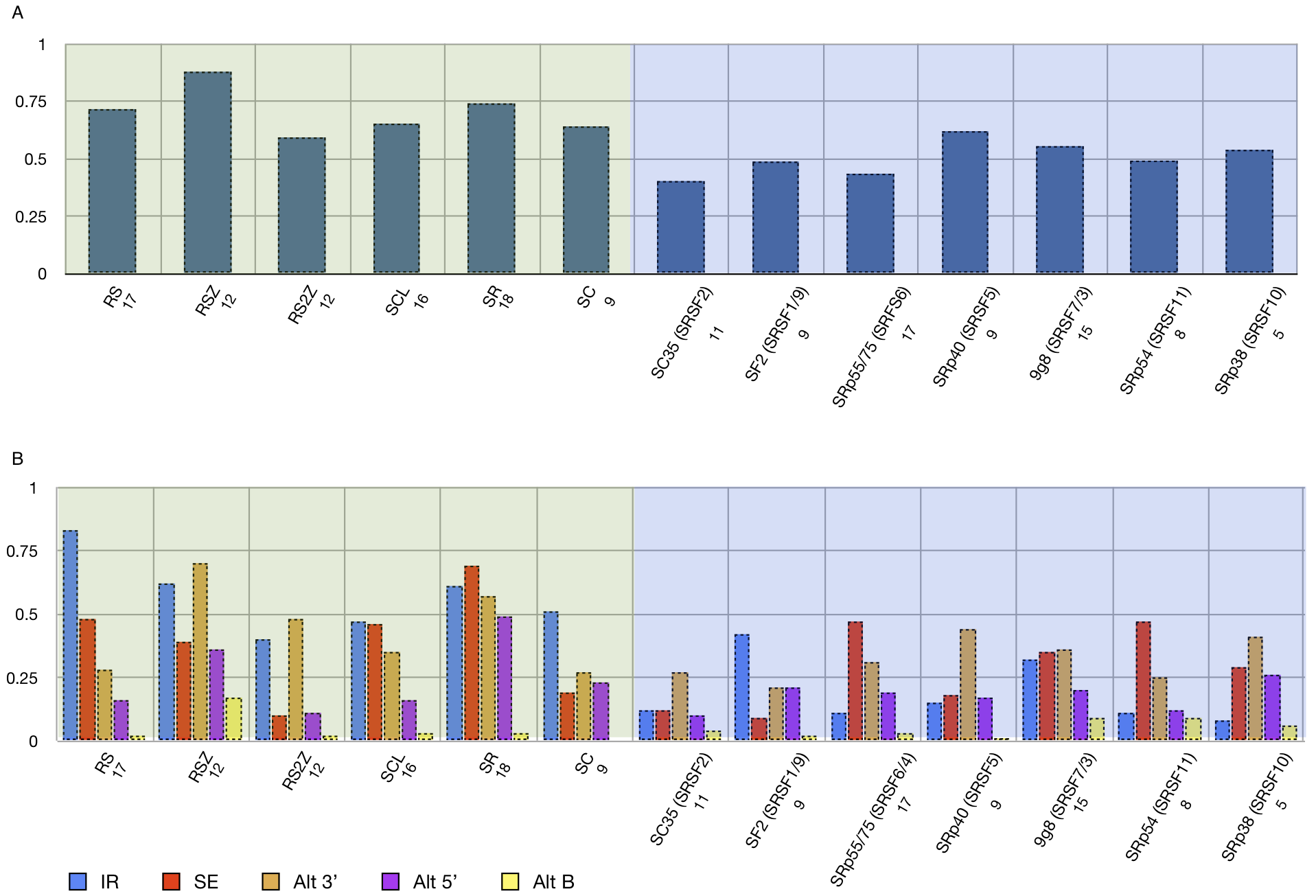

Supplement: Figure S11 — Family-wise AS comparisons. Panel A depicts the normalized proportion of genes undergoing AS per sub-family by averaging the values across the 100 trials in triplicate. Shading conventions are as previously described. Panel B shows the mean AS event type per gene experiencing AS in the normalization procedure but according to sub-family rather than organism (c.f. Figure S10). The Viridiplantae sub-families are shaded in green whereas the others are shaded in blue. The numbers below the sub-families designate the number of genes with AS in that particular sub-family. SRp40 and SRp55/75 are separated here to highlight differences between vertebrates and insects. (TIFF) [file pone.0024542.s011.tiff]

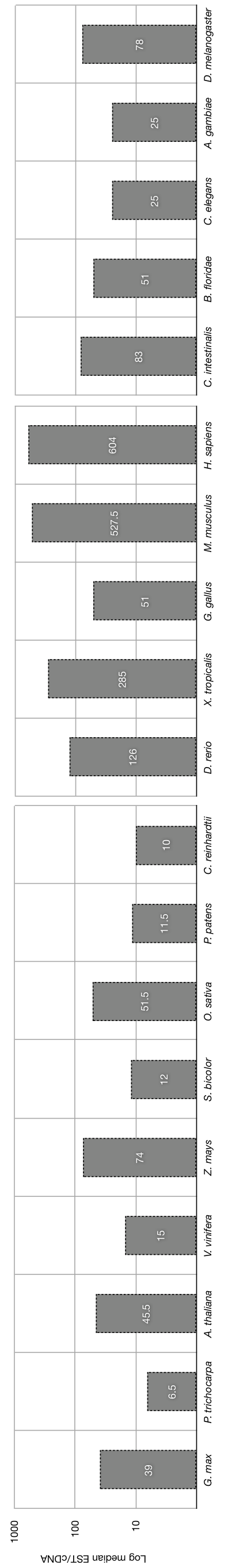

Supplement: Figure S12 — Log Median ESTs/cDNAs per organism. The median number of ESTs/cDNAs per gene per organism is presented on a log scale, with raw values indicated within the bars. (TIFF) [file pone.0024542.s012.tiff]

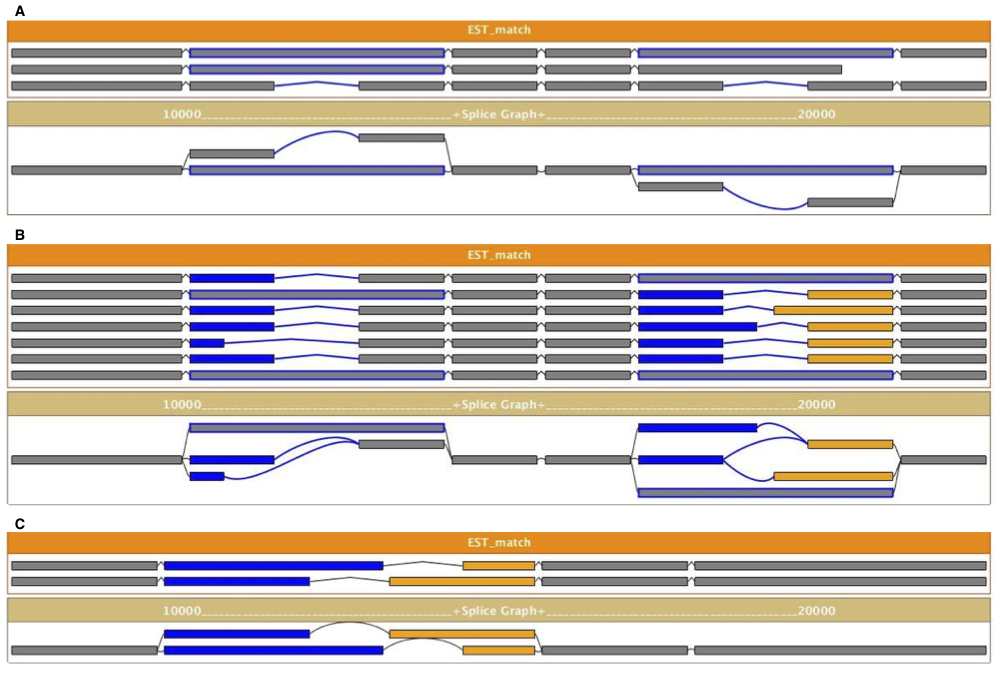

Supplement: Figure S13 — How AS event types are counted. As a simple example, consider the transcripts given in Panel A. Although there are two retained introns, the transcripts support only one intron retention event in which both introns are retained simultaneously. Consequently, for this graph we count a single intron retention event. A more complicated example is shown in Panel B. The graph has two retained introns for which three combinations are supported by EST transcripts. Additionally, there are two alternate 5′ events supported by transcripts and an alternate 3′ event. In this case, we count three intron retention events, two alternate 5′ events and a single alternate 3′ event. The rules for cassette exons are analogous to those for intron retention: when there is evidence of multiple skipped exons in a gene, we count number of distinct EST transcripts that support each combination. For alternative 3′ and 5′ splice sites, we use the most prevalent splice site (the one supported by a plurality of EST transcripts) and simply count the number of alternatives. When we cannot determine a prevalent form, we use the splice site that yields the longest intron. We distinguish between alternate 3′ sites (Alt 3′), alternate 5′ sites (Alt 5′) and simultaneous 3′/5′ events (Alt B). We count Alt B events whenever an alternative 5′ site is paired with the same alternate 3′ site in all transcripts. For example, in Panel C the alternate 3′ and 5′ splice sites are paired, so this will be counted as a single Alt B event. We incorporated our counting rules into our modified version of Sircah and generated statistics for each kind of AS event. (TIFF) [file pone.0024542.s013.tiff]
